# Contemporary epidemiological overview of malaria in Madagascar: operational utility of reported routine case data for malaria control planning

**DOI:** 10.1186/s12936-016-1556-3

**Published:** 2016-10-18

**Authors:** Rosalind E. Howes, Sedera Aurélien Mioramalala, Brune Ramiranirina, Thierry Franchard, Andry Joeliarijaona Rakotorahalahy, Donal Bisanzio, Peter W. Gething, Peter A. Zimmerman, Arsène Ratsimbasoa

**Affiliations:** 1 Center for Global Health and Diseases, Case Western Reserve University, Cleveland, OH 44106-4983 USA; 2 Department of Zoology, University of Oxford, Oxford, OX1 3PS UK; 3 National Malaria Control Programme, Ministry of Health, Androhibe, Antananarivo, Madagascar; 4 Department of Public Health, Faculty of Medicine, University of Antananarivo, Antananarivo, Madagascar; 5 Oxford Big Data Institute, Li Ka Shing Centre for Health Information and Discovery, University of Oxford, Oxford, OX3 7BN UK; 6 Faculty of Sciences, University of Antananarivo, Antananarivo, Madagascar

**Keywords:** Malaria, Madagascar, Routine health information systems data, Surveillance, Outbreaks

## Abstract

**Background:**

Malaria remains a major public health problem in Madagascar. Widespread scale-up of intervention coverage has led to substantial reductions in case numbers since 2000. However, political instability since 2009 has disrupted these efforts, and a resurgence of malaria has since followed. This paper re-visits the sub-national stratification of malaria transmission across Madagascar to propose a contemporary update, and evaluates the reported routine case data reported at this sub-national scale.

**Methods:**

Two independent malariometrics were evaluated to re-examine the status of malaria across Madagascar. First, modelled maps of *Plasmodium falciparum* infection prevalence (*Pf*PR) from the Malaria Atlas Project were used to update the sub-national stratification into ‘ecozones’ based on transmission intensity. Second, routine reports of case data from health facilities were synthesized from 2010 to 2015 to compare the sub-national epidemiology across the updated ecozones over time. Proxy indicators of data completeness are investigated.

**Results:**

The epidemiology of malaria is highly diverse across the island’s ecological regions, with eight contiguous ecozones emerging from the transmission intensity *Pf*PR map. East and west coastal areas have highest transmission year-round, contrasting with the central highlands and desert south where trends appear more closely associated with epidemic outbreak events. Ecozones have shown steady increases in reported malaria cases since 2010, with a near doubling of raw reported case numbers from 2014 to 2015. Gauges of data completeness suggest that interpretation of raw reported case numbers will underestimate true caseload as only approximately 60–75 % of health facility data are reported to the central level each month.

**Discussion:**

A sub-national perspective is essential when monitoring the epidemiology of malaria in Madagascar and assessing local control needs. A robust assessment of the status of malaria at a time when intervention coverage efforts are being scaled up provides a platform from which to guide intervention preparedness and assess change in future periods of transmission.

**Electronic supplementary material:**

The online version of this article (doi:10.1186/s12936-016-1556-3) contains supplementary material, which is available to authorized users.

## Background

**Fig. 1:**
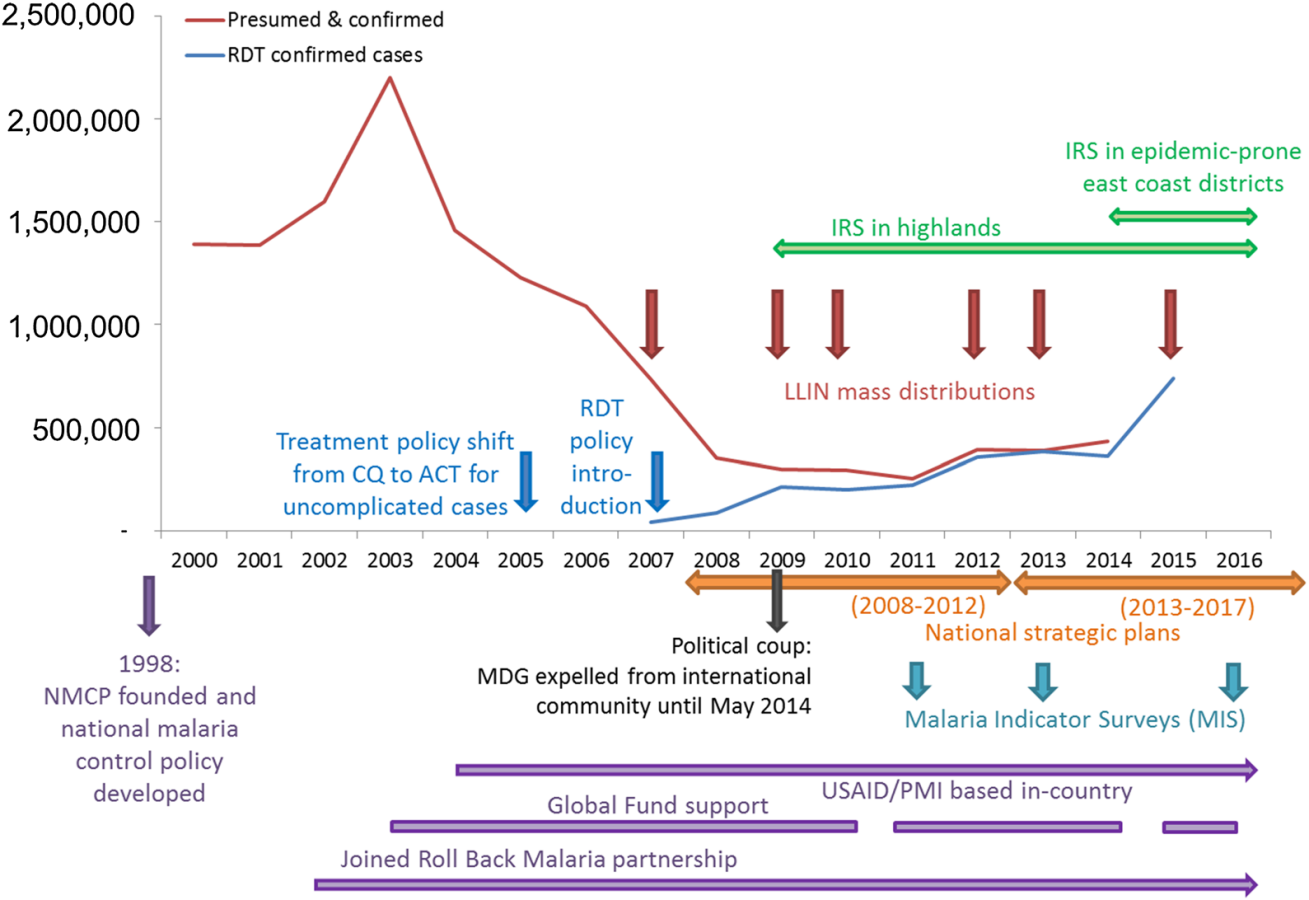
Timeline of milestones in malaria policy, funding, planning and implementation in Madagascar. Malaria cases (2000–2014) are plotted from data reported by the NMCP published in the WHO’s World Malaria Report 2015 [8]; numbers of RDT confirmed cases for 2015 are from NMCP directly. Intervals between Global Fund disbursement when no financial resources were available to the NMCP were: Sept 2010–Feb 2011; Sept 2014–March 2015; and July 2016 onwards (NMCP, pers. comm., June 2016)

The Malagasy context is one of protracted political instability, most recently in the aftermath of a political coup in 2009 which continues to bear severe economic and social impacts [1] (Fig. 1). A long-term lack of investment in the country’s infrastructure has had direct repercussions for malaria control efforts as well as the country’s broader developmental situation [2]. Madagascar has the seventh lowest per capita gross domestic product (GDP) globally [3] with an estimated 75.3 % of the population living under the national poverty line in 2010 [3]. This was estimated to have increased by more than 10 % since the 2009 crisis, with the World Bank estimating 92 % of the population to be living under $2 a day in 2013 [1]. The population lives predominantly in rural environments (66 %), and almost half (42.4 %) is younger than 15 years, and the median age is 18.4 years [3]. Malnutrition is rife, affecting over half of children, with 25 % of children reported to be severely malnourished [4], exacerbated by periodic famine outbreaks following severe drought [5] and failed harvests from locust infestation [1]. Childhood underweight is reported to be the primary overall risk factor of disease in the Malagasy population [6], increasing vulnerability to infections and development of severe sequelae. In 2012, top causes of consultations at health centres among under 5 year olds were, in descending order, acute respiratory infections, diarrhoea, digestive disorders, and uncomplicated malaria [7]. In the five to 14 years age bracket, uncomplicated malaria represented the second-most common cause of health centre consultations in 2012, while complicated malaria presented the top cause of district-level hospital mortality, at 10.1 % overall, and 21.6 % among under-fives [7]. All 23.9 million people across Madagascar’s 112 health districts are at risk of malaria exposure [8].

In view of this substantial disease burden, malaria represents a main priority for the Malagasy Ministry of Health (MoH) and associated international public health-focussed partners [9]. Intervention coverage is high, with some of the highest ownership rates of long-lasting insecticide-treated nets (LLINs) in the African region in 2013 [8, 10]. This was supplemented at the end of 2015 with the distribution of 12 million LLINs aiming at universal coverage of one net per two individuals across 92 of the country’s 112 health districts where seasonal or perennial transmission is considered to occur. In the lower transmission districts of the highlands, focalized indoor residual spraying of insecticide (IRS) has been deployed against the *Anopheles* vectors [11]. Nevertheless, increasing numbers of cases have been reported since 2010. This paper analyses the routine reported malaria rapid diagnostic test (RDT) data from 2010 to 2015 to evaluate trends of malaria transmission across Madagascar. This analysis provides a benchmark assessment of malaria endemicity ahead of the anticipated impact of the large-scale LLIN distribution campaign prior to the 2016 transmission season. December 2015 also represented the end of the Millennium Development Goals era (2000–2015) and Fig. 1 summarizes the malaria control activities that have contributed to the malaria-specific aspects of this initiative. This overview therefore comes at an opportune time to assess progress against the target of “reversing the incidence of malaria” in Madagascar [12, 13].

Transmission of malaria is heterogeneous across Madagascar [14]. The island, the fourth largest globally at over 1500 km in length and with a surface area of 587,000 sq km, similar to California or Sweden, is highly ecologically and climatically diverse [15, 16]. Reflecting these conditions, the epidemiology of malaria across the island shows distinct seasonal trends and transmission intensities between regions [17, 18] with different vector species and behaviours predominant in different areas [19, 20]. Madagascar has a stated aim to reduce the proportion of malaria-attributable fevers and mortality, and start transitioning towards pre-elimination status by end of 2017, the end of the country’s current National Strategic Plan [21] (Fig. 1). The development of spatially specific interventions informed by local transmission characteristics is therefore required to optimize the efficacy of strategies to combat malaria.

**Fig. 2:**
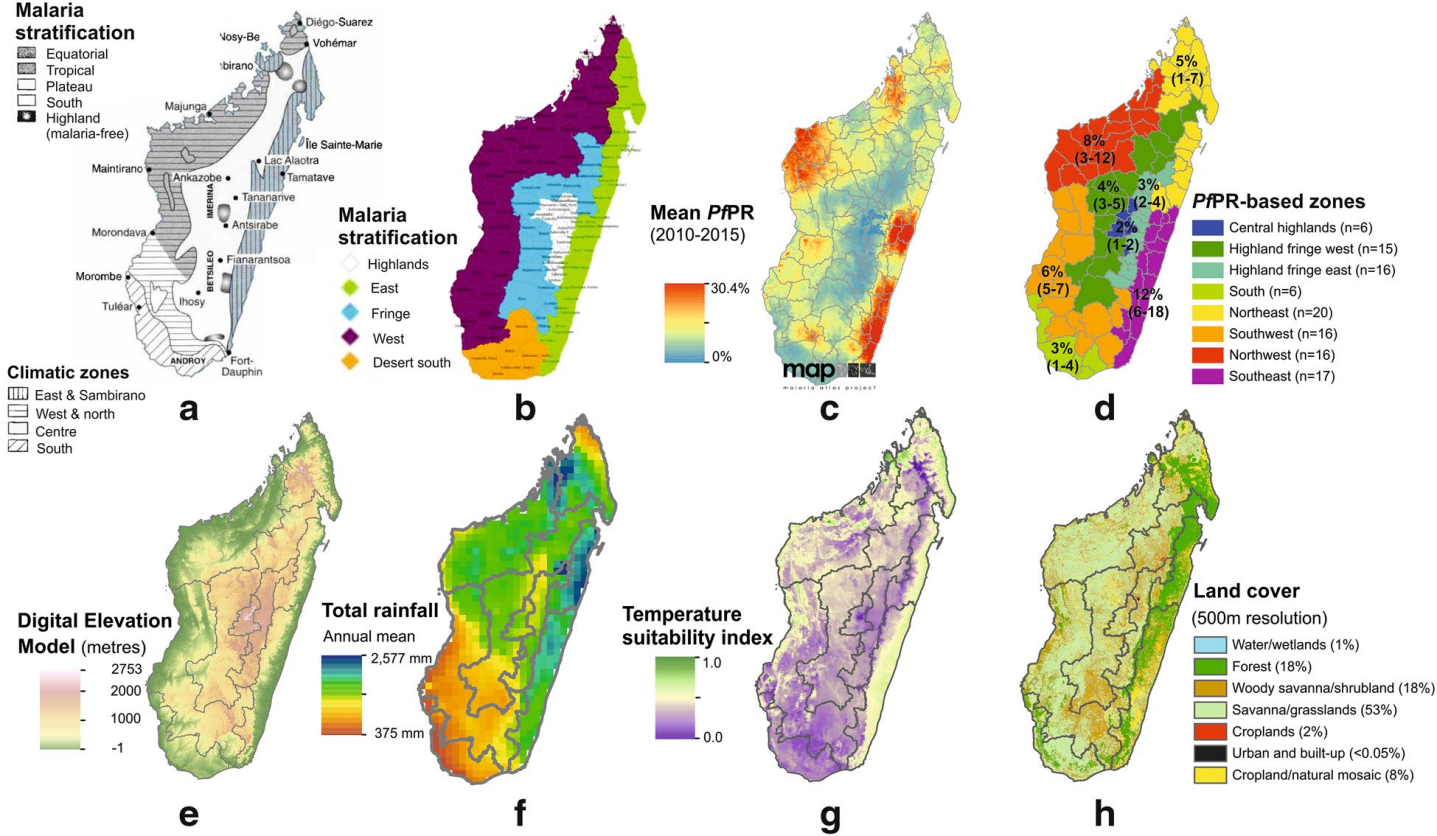
Defining a contemporary sub-national stratification of malaria transmission intensity in Madagascar. **a** Mouchet et al.’s stratification of malaria transmission across Madagascar (reproduced with permission [22, 23]); this map was adapted to district-level administrative boundaries and **b** shows the currently used adaptation of the sub-national stratification (Source: NMCP National Strategic Plan [21]). **c** Represents the Malaria Atlas Project (MAP) modelled map of *P. falciparum* prevalence in 2–10 year olds (*Pf*PR mean surface from 2010–2015) [14], and **d** the updated ecozones based off the *Pf*PR map (regional figures correspond to the mean district-level *Pf*PR value (2010–2015), and the min–max *Pf*PR values from the districts in each ecozone. **e**–**h** Illustrate some of the environmental covariates which informed the *Pf*PR mapping model, and which therefore underpin the updated sub-national stratifications. **e** A model of digital elevation (source: Shuttle Radar Topography Mission (SRTM) [59], plotted at 1 km resolution). **f** Maps mean total annual rainfall (mm) from 2010 to 2014 (source: NASA Tropical Rainfall Measuring Mission (TRMM) 3B43 algorithm available at 0.25º by 0.25º spatial resolution), while **g** shows the annual temperature suitability for *P. falciparum* transmission, reproduced with permission from Weiss et al. [60]. **h** Summarizes land-cover use across Madagascar at 500-m spatial resolution (IGBP MODIS annual landcover product MCD12Q1 [61, 62])

Mapping the intensity of malaria transmission across the island has been done since the start of control efforts. Joncour et al. published district-level endemicity estimates according to splenic rates in the 1950s, identifying broad categories of malaria transmission [18]. These were updated by Mouchet et al. in the early 1990s, stratifying the country according to climatic and transmission patterns (Fig. 2a [22, 23]). These mapping sub-divisions have subsequently adapted to changing needs, aligning with contemporary administrative boundaries (Fig. 2b [21, 24, 25]), and recently been further simplified from Mouchet’s five zones to two broad zones representing high transmission (east and west coasts) and low transmission (highlands and arid south) by control programme agencies [11, 21, 25]. Here, the boundaries of these sub-national divisions are re-evaluated and used to describe the contemporary epidemiology of malaria in Madagascar more than a decade after the country started receiving substantial external funding to roll-out its control programme (Fig. 1).

## Methods

Two methodological steps were followed to address the paper’s goal of assessing Malagasy routine health facility data and its application to programmatic planning. First, regional sub-divisions were reviewed and updated in accordance with contemporary transmission patterns. Second, routine health reports were examined within the updated sub-national stratifications to identify each area’s key epidemiological characteristics which might impact on intervention planning. The data sources accessed in this study are summarized in the Additional file 1.

### Sub-national stratification of malaria transmission

Transmission intensity, quantified as the community-level prevalence of blood stage infection, has been a traditional marker for regional stratification to guide malaria control [26, 27]. This approach bypasses potential weaknesses associated with the surveillance and health reporting systems. In this present study, mean summary maps of the modelled prevalence of *Plasmodium falciparum* infection across the 2–10 year old age range (*Pf*PR) from the Malaria Atlas Project (MAP; [14, 28]) were used to assess the regional patterns of malaria infection (Additional file 2). These maps are informed by an extensive range of malariometric (*Pf*PR community surveys from Malaria Indicator Surveys [24, 25] and all other available surveys e.g. [29], as well as annual case incidence estimates), intervention coverage [10] and environmental covariate datasets [30]. For the present paper’s objectives, the mean of the 2010–2015 annual surfaces was calculated (*Pf*PR_2010–2015_; Additional file 2; Fig. 2c). This timeframe ensured consistency with the health facility case data that were available for investigation in this paper and provided a contemporary picture whilst attenuating inter-annual fluctuations in the *Pf*PR estimates. For operational utility, the spatially continuous mean *Pf*PR_2010–2015_ map was aggregated to current sub-national political boundaries, and then used to sub-divide the country into ecozones of contiguous districts with similar transmission intensity (Fig. 2d). The aim of the stratification was to generate an output that was pertinent for operational purposes and that allowed an intuitive description of the sub-national epidemiology of malaria. The number of ecozones was not prior constrained though the aim was to keep this under ten to ensure programmatic ease and avoid too much duplication across zones in terms of overlapping epidemiology. Automated clustering was attempted using packages in spatial analysis software but these were not well adapted to this present analysis due to the range of mean district level *Pf*PR_2010–2015_ values which meant that the clustering analysis mainly identified single isolated outliers without also encompassing the practical aspects of operational utility. The final stratification approach was therefore driven by two objective criteria including (i) spatial contiguity for operational convenience and (ii) transmission intensity as determined by the district-level mean *Pf*PR_2010–2015_ map. Some subjective decision-making was necessary in situations when these two criterion could not both be met and instead one needed to be prioritized over the other. Specific examples are given in the Results.

### Routine health facility data

Routine health management information system (HMIS) data reports from January 2010 to December 2015 were evaluated to assess the characteristics of the evidence-base of case reports available to inform decision-making by the Madagascar National Malaria Control Programme (NMCP). The routine HMIS in Madagascar follows a bottom-up pyramid, multi-step process of data aggregation and transfer between levels according to a fixed monthly schedule. Paper-based summaries of all patient consultations at the community-level health centres, either at health facilities or through community health workers, are transferred to the 112 health district offices across Madagascar where data are entered into the database software GeSIS, which is accessible by the regional and central MoH, including the NMCP [31, 32]. Private clinics are on the periphery of the HMIS but are intended to report to the public health system though many do not, so these are acknowledged as being poorly represented in the routine data system [21].

The Malagasy NMCP does not distinguish between species of malaria in its reporting systems. Combination RDTs are used which can distinguish *Plasmodium falciparum* from other species [33, 34], but this information is not recorded. The clinical cases described here therefore refer indiscriminately to any species of human *Plasmodium* infection, though cross-sectional surveys have indicated that infection is predominantly from *P. falciparum* (>99 % by PCR when aggregated nationally [24]).

### Outbreak surveillance and definitions

Alongside the HMIS, a parallel data reporting channel, the Integrated Diseases Surveillance and Response System, is intended as an outbreak early-warning system for notifiable diseases of epidemic potential, including malaria. Paper or electronic reports are sent weekly to the central NMCP for analysis. In practice, this reporting channel is weak and poorly developed in most health districts; the US President’s Malaria Initiative in Madagascar identified only eight of 112 districts as having functional reporting [11]. Outbreak surveillance is also conducted directly at the health facility level based on weekly trends in observed RDT-positive case numbers. Other surveillance institutions also act as channels to alert NMCP of outbreak situations, such as the Institut Pasteur’s nationally distributed sentinel site network [35, 36].

The NMCP respond to two categories of outbreaks [21]. In areas considered at very low risk of malaria, notably the Central highland and desert South ecozones, evidence of autochthonous transmission triggers an emergency response. In higher transmission zones outbreaks follow epidemic trends, where escalation of case numbers is commonly associated with mortality. In these latter areas, two operational definitions of outbreaks are applied at the health-facility level which are consistent with approaches followed elsewhere [37, 38]: (i) a weekly total of RDT-confirmed cases exceeding a pre-defined weekly health centre-specific threshold (calculated as two standard deviations from the mean number of confirmed cases from at least the preceding 3 years of data); or, (ii) in health centres lacking a defined outbreak threshold, a weekly doubling of RDT-positive cases over 3 consecutive weeks [21, 39].

Outbreak reports from January 2012 to December 2015 were collated from NMCP surveillance records to assess district-level temporal and spatial trends in outbreak occurrence based on these definitions.

### Demographic data

To ensure consistency with NMCP estimates, official demographic data from the Madagascar MoH [40] were used in incidence calculations. As per the MoH protocols, a fixed annual population growth rate of 2.8 % was applied cumulatively at the district level to population data collected from the last official national census in 1993. In the absence of more specific population age distribution data, the MoH also defines standardized age categories, with children under 5 years representing 18 % of the population total. This age bracket differs from the age categories in the malaria case reporting database which reflects anti-malarial treatment dosing age categories, so includes 5 year olds with under-fives. To account for this disparity, the proportion of 5 years old within the 5–14 years category was estimated (28.6 % of the overall population, thus 2.86 %). This therefore corresponded to an estimated 20.86 % of the total population being under 6 years [40].

### Proxy indicators of data reporting completeness

Indicators of reporting completeness, including the numbers of health centre reports received at the central level and the proportion of distributed RDTs accounted for in the health centre reports, were obtained from MoH records through the NMCP. The 2014 database of drug and RDT stock-outs, which reported the cumulative total days of consumable stock-outs annually, contained blank entries. Stock-out reporting required active participation by each health centre, thus the lack of reporting could not necessarily be interpreted to indicate ‘zero stock-outs events’. To address this characteristic of the dataset, a conservative approach was followed, whereby health centres that reported no specific value for RDT stock-out events but did report on the status of other consumables, was taken to indicate zero stock-out days of RDTs. Health centres that did not report at all were excluded from this analysis.

Related variables, including the proportion of the population resident further than 5 km from a health facility and district office accessibility from health centres, were also collated and evaluated.

## Results

### An updated map to reflect the contemporary stratification of malaria transmission

The MAP’s *Pf*PR map predictions for 2010–2015 (Fig. 2c; [14]) were summarized to the district level and examined for regional trends (Additional file 2). Figure 2e–h illustrate a subset of the main environmental covariates used in the *Pf*PR modelling process. The overall range of district-aggregated mean *Pf*PR values was relatively narrow, with only 17 districts exceeding 10 % (these summary metrics mask sub-district variation). This therefore limited the applicability of the traditional malaria endemicity categorization, which has 10 % *Pf*PR as the upper threshold of its lowest endemicity category: ‘hypoendemic’ [27]. Instead, narrower intervals were necessary to distinguish local transmission intensity patterns. Overall, eight distinct transmission zones emerged from the *Pf*PR_2010–2015_ output map (Fig. 2d). Ensuring spatially contiguous zones meant that some overlap in the ranges of mean *Pf*PR_2010–2015_ values was necessary. In different areas, it was sometimes necessary to prioritise differently the two stratification criteria of (i) spatial contiguity and (ii) local transmission intensity similarities. For example, the east coast districts of Mananjary and Ifanadiana (approximately two-thirds down the east coast) had lower *Pf*PR_2010–2015_ than the neighbouring east coast districts. However, to ensure spatial contiguity in the ecozones, these two isolated districts were included in the Southeast ecozone. Moreover, these two districts showed greater similarity in terms of transmission intensity to the Southeast districts than to the Highland fringe districts. In contrast, the high altitude central highland areas include small islands of very low/negligible autochthonous transmission. The unique epidemiology in these areas justified their amalgamation as a single ecozone despite lacking spatial continuity as they would require distinct intervention planning and therefore distinct ecozone classification. Similarly, Maroantsetra district was classified into the Highland fringe west ecozone due to its transmission characteristics being more similar to those than to the Northeast ecozone districts with which Maroantsetra shared north and south borders. The spatial continuity of the Northeast ecozone was not considered broken by this as the resulting Northeast ecozone included the full northern section of the east coast. The stratification therefore aimed to be objective but nevertheless included some subjective decision-making to optimise the final product for operational utility.

Relative to previous stratifications (Fig. 2a, b), the new *Pf*PR-based stratification (Fig. 2d and tabulated in Additional file 3) uses a larger number of classes (eight versus four to five in previous versions). The eight zones came about directly from the dataset, the number was not pre-determined. These additional divisions mean that greater resolution in the epidemiology of each zone can be characterized. Areas at each extreme of the transmission spectrum are more clearly differentiated from neighbouring zones. For example, the previous single ‘east coast’ zone is now split into two areas of quite distinct transmission characteristics across the northern and southern east coast districts, allowing the high transmission areas to be more clearly identified.

### Trends in routine malaria case reports

**Fig. 3:**
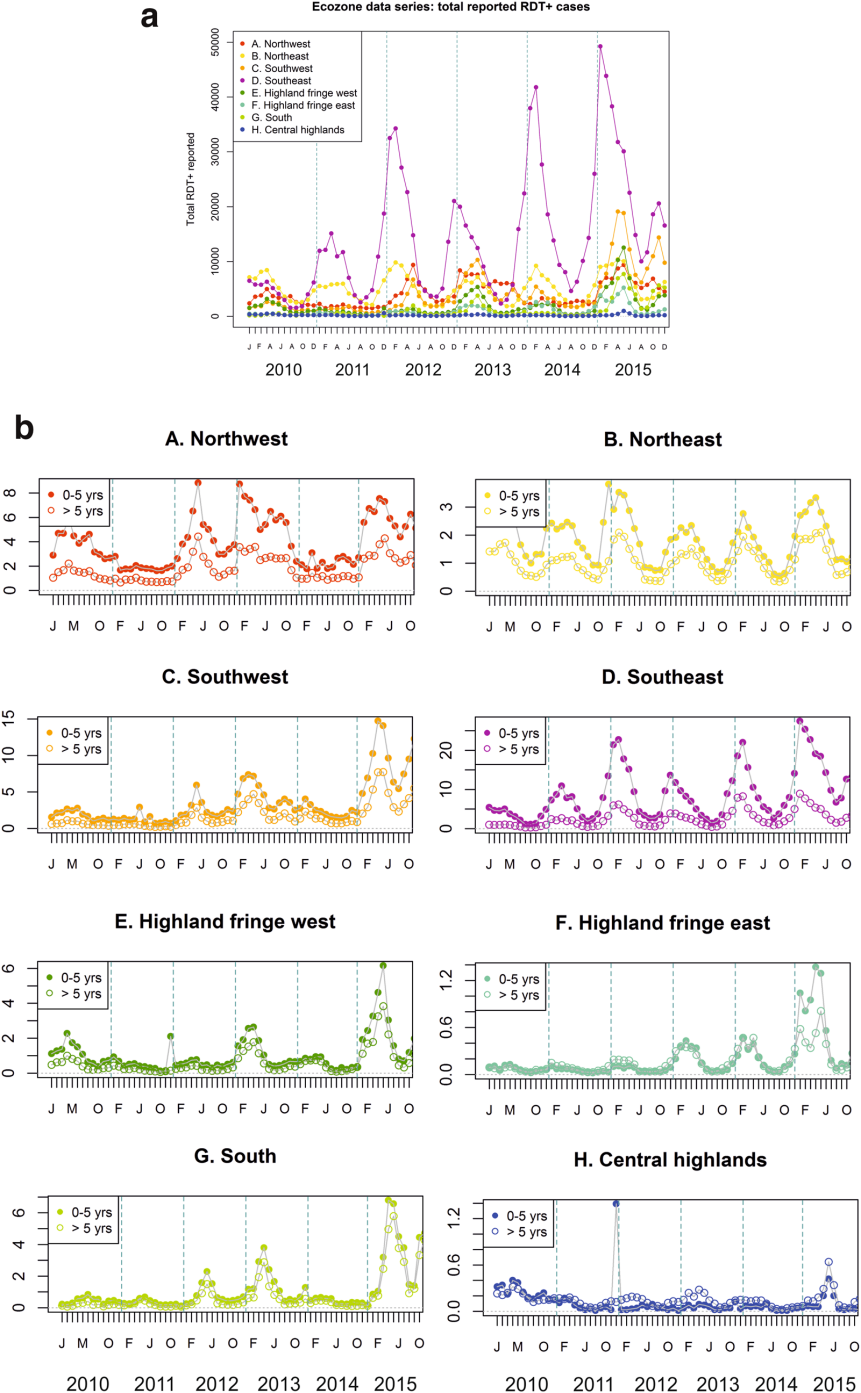
Descriptive plots of the RDT-confirmed case data available in the NMCP database for 2010–2015. **a** Raw reported confirmed case numbers in the NMCP database, aggregated monthly into the eight ecozones. Additional plots including individual ecozone plots are available in Additional file 2. **b** Raw reported age-specific monthly incidence of RDT-confirmed cases per 1000 population. Population size is estimated annually based on a fixed growth rate (see “Methods” section). All-age population estimates for 2015 by ecozone are (Ministry of Health, 2015): *A* Northwest: 1.907 million; *B* Northeast: 4.470 million; *C* Southwest: 2.083 million; *D* Southeast: 3.855 million; *E* Highland fringe west: 2.907 million; *F* Highland fringe east: 5.759 million; *G* South: 1.298 million; *H* Central highlands: 1.659 million. Additional plots and tables relating to this data set are included in Additional file 2

A total of 175,061 health facility reports were available for analysis which had been reported from Madagascar’s 3924 health facilities to the central NMCP over the 2010–2015 time frame. During that time, absolute numbers of reported RDT-confirmed malaria cases almost quadrupled to a maximum in 2015 of 738,996 reported cases, a steady increase from 2010 when 201,135 cases were reported to the NMCP (Fig. 3a; Additional file 4A). The greatest year-on-year increases were between 2011–2012 and 2014–2015, when there were 65 and 97 % increases (i.e., near doubling) of reported cases, respectively. All ecozones other than the Central highlands reported increases of reported cases between 2010 and 2015. In the Northeast, there was only a 30 % increase (37,884–78,716 cases), while in other zones the reported increase ranged from doubling to tenfold increases (Fig. 3a). The recent 2014–2015 increase in reported cases was evident from all zones, with five zones reporting more than a doubling of cases in that 1 year period and the South ecozone reporting a tenfold increase from 4238 reported cases in 2014 to 47,583 in 2015. The Southeast ecozone had a 41 % increase in reported cases from 2014 to 2015 (218,675–308,192 cases), accounting for just under half (42 %) the country’s total reported cases for 2015 despite having only 16 % of the country’s population [40]. The Southeast ecozone was the only area to have also reported a substantial increase in case numbers from 2013 to 2014 (66 % increase), other areas had seen a decrease or only very slight increase ahead of the major increases into 2015 (Fig. 3a). Seasonal trends are largely consistent through the time window evaluated, peaking in April–May except in the two east coast ecozones where an earlier February peak is generally observed. Trends in the lower endemic Central highlands are less consistent. Additional file 4A–D provides a more detailed analysis of the temporal structure of the data through autocorrelation plots of the time series, and linear regression and generalized additive models.

Incidence of reported RDT-confirmed cases nationally peaked for 2010–2015 in April–May 2015 at 3.8–4.0 cases/1000 population/month, having increased significantly since 2010, increasing from annual incidence of 9.65/1000 RDT+ cases in 2010 to 30.87/1000 reported in 2015 (linear regression model p < 0.001; Additional file 4B). As detailed in Additional file 4B, the fastest rates of increase in incidence were reported from the southern ecozones: the Southeast, Southwest and South (in descending order) had the sharpest rates of increase. Negative trends were identified from the Northeast and Central highland ecozones, indicating a decrease in incidence, though these trends were non-significant (linear model p > 0.05). Monthly incidence across two age categories (0–5 and >5 years; Fig. 3b) revealed significantly different age-specific incidence in all ecozones except the Highland fringe east (Wilcoxon Signed-Rank test with 95 % confidence). The Central highland ecozone was the only area where the incidence of malaria in over 5 years olds exceeded that of infants (0–5 years), in all other ecozones where there was a significant difference, the caseload was greater in infants under-five (Fig. 3b). This was particularly marked in the Southeast, where the under-five incidence was on average 3.7 times greater than in the older age category (range: 2.3–5.5).

To account for the potential distortion of temporal trends in raw reported numbers resulting from incomplete reporting or diagnostic kit stock-outs, RDT-confirmed case counts were adjusted to the total reported number of consultations and the overall number of reported RDT results (Additional file 4C–D). HMIS reported consultations increased by 51 % from 4.8 million in 2010 to 7.3 million in 2015. During the same time period, total reports of diagnostic tests performed more than doubled from 603,727 to 1,487,745. Trends in reported RDT use closely reflect the temporal trends in confirmed case reports (Additional file 4D). Trends in these adjusted datasets were less apparent than from the raw reported data, but linear regression models still indicated overall increasing burden in both adjusted metrics nationally. Across the country, the annual diagnostic test positivity rate increased steadily from 33 % in 2010 to 50 % in 2015, indicating that despite substantial increases in the use of diagnostic testing, there was nevertheless an increase in the proportion of fever cases seeking treatment that were positive for malaria. The increasing burden of malaria was particularly apparent in the south where the change in RDT positivity rate increased significantly over the 6-year reporting period in the Southwest (35 % in 2010 to 60 % in 2015), Southeast (39 to 60 %), South (16 to 58 %), and both Fringe (west: 28 to 41 %; east: 9 to 24 %) ecozones (p < 0.05 by linear regression; Additional file 4D). Other zones had a much smaller effect size of change [negative for the Northeast (37 to 35 %) and Central highlands (17 to 15 %)], which was non-significant. In the Southwest, positive malaria diagnoses were relatively rare before 2013, with only 3 months reported to have >10 % of consultations attributable to malaria; in contrast, health centre reports from 2013 to 2015 indicated 25 months to have had >10 % of consultations concluding with an RDT+ diagnosis (Additional file 4C). In the higher-endemic Southeast, health facility reports noted only 2 months during 2010 when RDT+ consultations were greater than 12 % of all consultations; in contrast, this occurred during all months of 2015. Seasonal trends from these reporting-adjusted figures are less prominent than from the raw RDT positive counts (Fig. 3a), but nevertheless apparent.

An annual lag in autocorrelation across the time series of diagnostic positivity rate was particularly apparent from the east coast ecozones (Northeast, Southeast and Fringe areas), indicative of a seasonality effect, though this was less evident from western areas (Additional file 4D).

### Completeness of the HMIS data

**Fig. 4:**
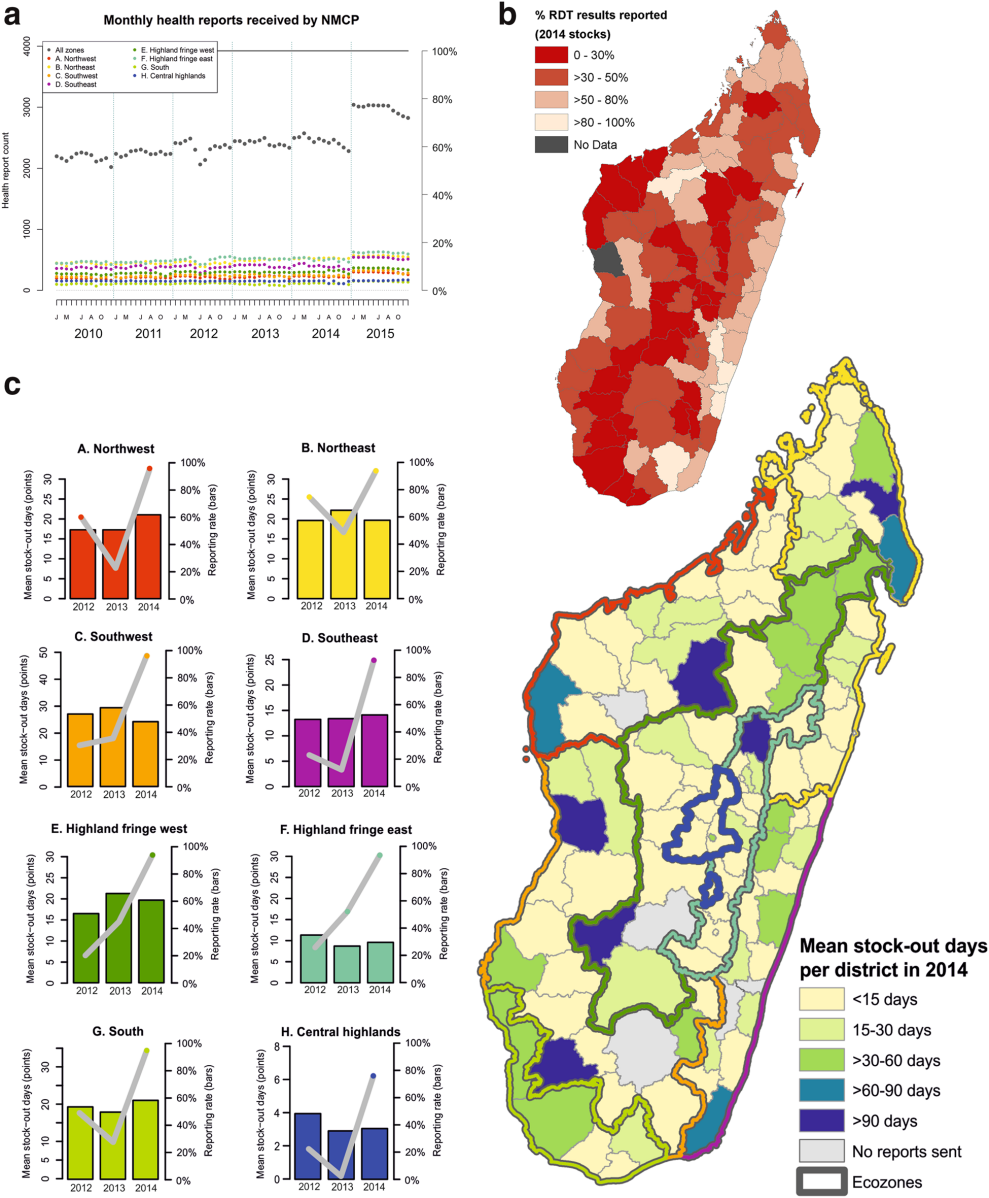
Indicators of the completeness of routine HMIS reporting. **a** Monthly health centre reports received by the NMCP. The *solid black line* indicates the total number of health facilities in Madagascar (n = 3924 [41]). The *right-hand axis* refers to the % of overall health centres reporting across all zones. Data represent a monthly report being submitted, but provide no indication of the completeness of the submitted reports. **b** Proportion of distributed RDTs for which a result (either positive or negative) was reported to the HMIS in 2014. Overall, 1.86 million RDTs were distributed to health districts nationally, and results for 0.94 million (50.5 %) were reported into the HMIS database. **c** Reported RDT stock-out incidence. The map shows the district-level mean RDT stock-out days in 2014. Only data from health centres that submitted at least one report for drug/diagnostic availability in the year were included in the mean calculation. Plots show stock-out status from 2012 to 2014 per ecozone. Points correspond to the mean number of stock-out days annually (*left hand y axis*) and *bars* represent the reporting rate within each ecozone (*right hand y axis*)

Of the 3924 health facilities intended to contribute monthly reports to the HMIS in 2015 [41], 214 were hospitals or specialist institutions, 2563 were community-level public health facilities staffed by either doctors (63 %) or nurses (37 %), and 1147 were private-sector institutions. Reporting improved across the 6-year period reviewed, from a mean monthly reporting rate of just over half of health facilities (55 %) in 2010, increasing to three-quarters (76 %) in 2015 (Fig. 4a). Some inter-annual fluctuation in total facility count is anticipated due to facility closures and openings, but fluctuations in the numbers of received reports are likely to be principally attributable to under-reporting. An additional source of malaria case data comes from the network of community health workers (CHW) active across the country [41, 42]. However, these volunteers report their activities through their health facility, not directly to the HMIS. Increasing numbers of active CHW should not affect the number of monthly reports submitted to the HMIS, though it will impact on the numbers of fever-related consultations performed.

While Fig. 4a data indicate that a report was received at the central level from the health facility, it provides no indication of the quality of the report. A proxy indicator of reporting completeness may be represented through evaluation of the proportion of RDTs distributed for which a result was reported into the NMCP database (Fig. 4b). Important limitations exist to assuming this proxy: not necessarily 100 % of RDTs distributed would be needed within the year, RDT distribution may not be constant through time (some health facilities may receive large deliveries some years and carry lower stocks in other years), and the risk that not all distributed RDTs are fit for use (some may be discarded due to void test results or overdue expiry dates). Nevertheless, the broad categories used in Fig. 4b indicate that even making allowance for distributed RDTs remaining unused in the health centres, 79 of 112 health districts (71 %) had more than half of their distributed RDT stock results in 2014 unaccounted for in the HMIS; in 34 districts, reports were received for fewer than 30 % of the distributed RDTs. Overall, 50.5 % of the distributed RDTs across Madagascar in 2014 (n = 1.856 million) were accounted for in the HMIS results. When aggregated to the ecozone scale, reporting from Southeast ecozone was highest, accounting for 78 % of distributed RDTs, while the South ecozone had poorest reporting, accounting for only 24 % of distributed RDTs in 2014. Rates in all other ecozones ranged between 29 and 56 %.

Case management policies in Madagascar require RDT confirmation for a case to be treated with anti-malarials and counted as a confirmed case in the reporting system. RDT stock-outs will therefore impact directly on the numbers of cases reported. Reporting of presumed cases is not consistently distinguished from confirmed cases in the monthly reports, introducing uncertainty into the reports (NMCP, pers. comm.). Nationally in 2014, 51 % of health facilities sent no reports of any stock inventories (including of drug stocks), while those that did report indicated a mean number of 30.5 days with RDT stock-outs (range: 0–330 days). Figure 4c summarizes the district stock-out data for 2014 (map) and reporting characteristics by ecozone from 2012 to 2014 (graphs). Bar plots (right hand y axis) show the health centre reporting rates over time, indicating the large numbers of health centres failing to report, and thus the difficulty with interpreting the data on numbers of stock-out days, which otherwise indicate that stock-outs are relatively infrequent events.

**Fig. 5:**
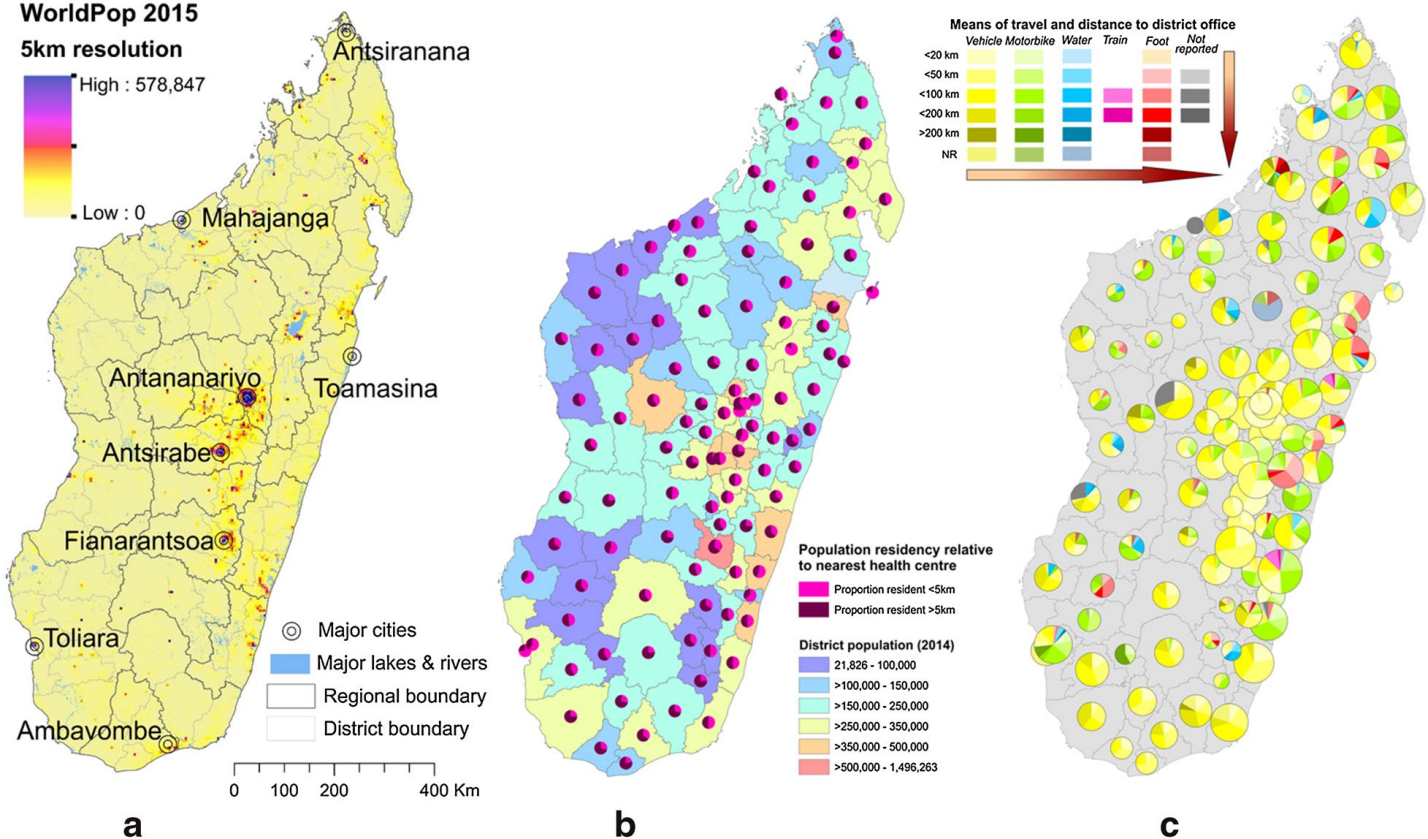
District-level summaries of health centre accessibility, **a** population density in 2015; **b** health centre accessibility to catchment populations and **b** health centre accessibility to the health district headquarters where monthly data must be reported. **a** Maps population density in 2015 (WorldPop [63]) together with cities estimated to have populations >100,000). **b** Shows the proportion of the population resident more than 5 km from their nearest health facility (equivalent to further than approximately one h walk). The *underlying colour* indicates the total population resident in the district, while the pie charts show the proportion of the population per district resident further or closer than 5 km from their nearest health facility. This is based off a subset of 2690 health facilities (69 % of all health facilities) for which data were available. **c** *Maps pie charts* representing different categories of accessibility for each health centre per district. *Colours* represent the available mode of transport, while colour intensity indicates distance categories between health centres and the health district headquarters. *Pie chart sizes* indicate the number of health centres needing to report data per district (range: 3–55). *Arrows* indicate the increasing difficulty with reporting. Data from the NMCP (Fig. 5b) and the Malagasy Health Sector Development Plan for 2014-2019 [64] (Fig. 5c)

Health centre isolation was evaluated as a potential factor contributing to reporting incompleteness (Fig. 5). Figure 5a maps population density across the country, while Fig. 5b charts the proportion of each district’s population living within 1 h walk of their nearest health facility (5 km). The colour index in Fig. 5c indicates the relative difficulty for health centre staff to access the health district office to submit their monthly health report. Overall, 56 % of the population are reported to live further than 5 km from their nearest health centre. This was generally consistent across all ecozones (range: 51–64 %), peaking in the Southwest. Nearly half of health facilities were situated more than 50 km from their district office (1125 of 2690 health facilities for which location details were available), with 13 % (n = 147) of those accessible only by foot or river/sea.

Health centre isolation data (quantified at the district level as the mean travel time from each health centre to the central district office) were analysed for potential correlations with 2014 data on: (i) RDT result under-reporting; and, (ii) the RDT stock-out reporting rate. No correlation emerged between these datasets, which were only available aggregated to the district level.

### Outbreaks

**Fig. 6:**
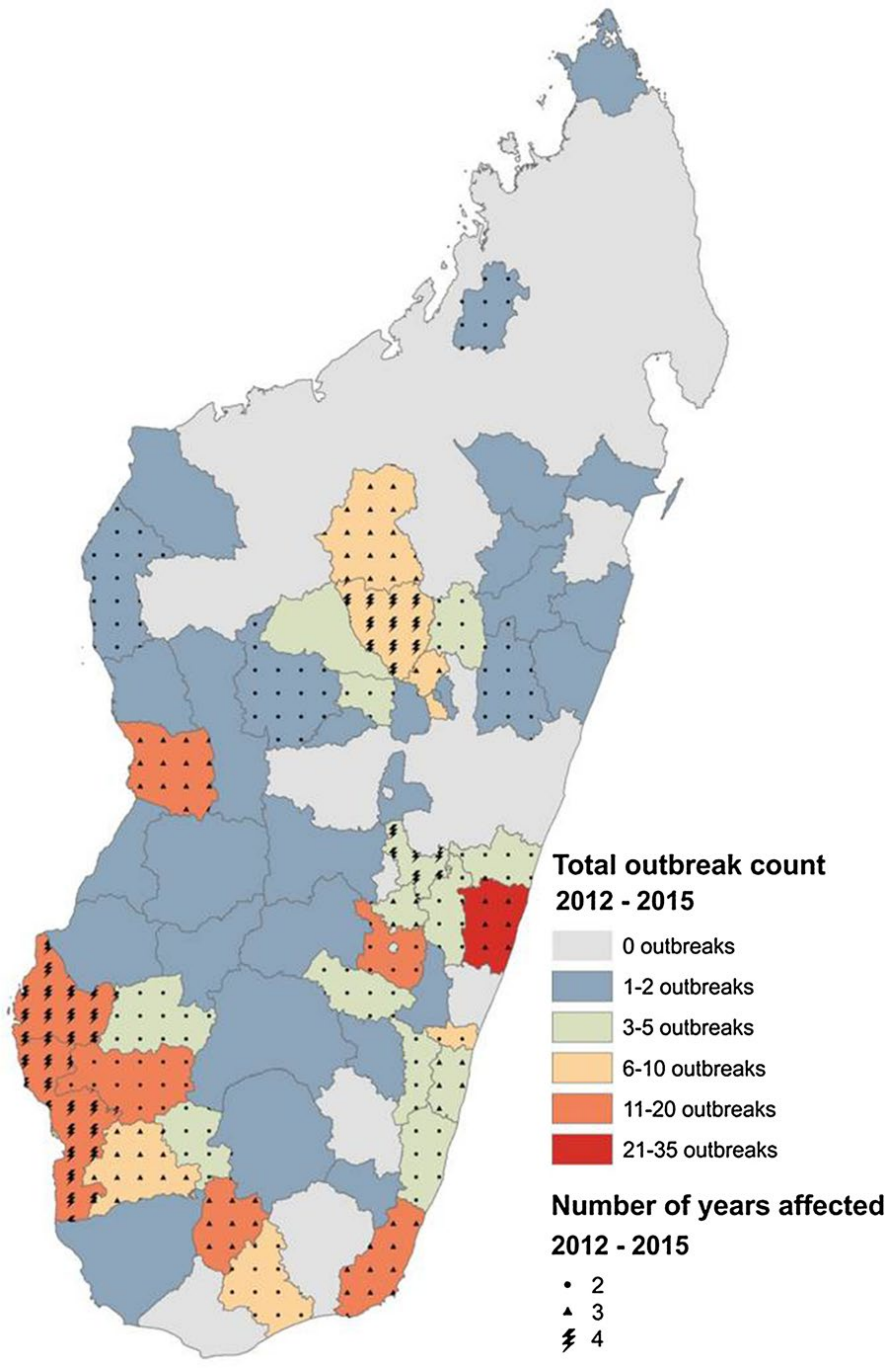
Outbreaks documented in the NMCP records for January 2012–December 2015. Outbreak definitions vary according to expected local transmission characteristics. *Colours* indicate the total number of outbreaks reported per district during the period examined. *Overlain symbols* indicate the number of years affected between 2012 and 2015. *Grey background* indicates no reported outbreaks

The nature of transmission events classified as ‘outbreaks’ differs substantially between affected geographic areas, as previously defined. Nevertheless, here these are considered together, irrespective of the particular outbreak types. This was partly due to data availability on the classifications of outbreak events, but also because all types of outbreaks require NMCP intervention, irrespective of the circumstances. Overall, therefore, January 2012 to December 2015 yielded a total of 292 outbreaks reported to the NMCP’s surveillance system as shown in Fig. 6 (instances of autochthonous transmission in low transmission ecozones, or an unexpected excess of cases in endemic areas; definitions provided in “Methods” section). Total reported outbreaks per year were 42 in 2012, 107 in 2013, 60 in 2014, and 83 in 2015. Over half (n = 63) of the country’s 112 districts reported an outbreak during this period; 31 of these reported more than one outbreak. Overall, half the total reported outbreaks occurred in just ten districts. At the commune level (administrative sub-division of a district), 193 of 1579 communes were affected by outbreaks between January 2012 and December 2015 (12.2 %), though 73.1 % of these reported only a single outbreak. Only 7.3 % (n = 14) of affected communes reported more than two outbreaks during the 4 year reporting period examined. The data suggest that although districts could be categorized as being at higher or lower risk of epidemics, at the commune-level outbreaks do not appear to have a strong temporal association.

**Fig. 7:**
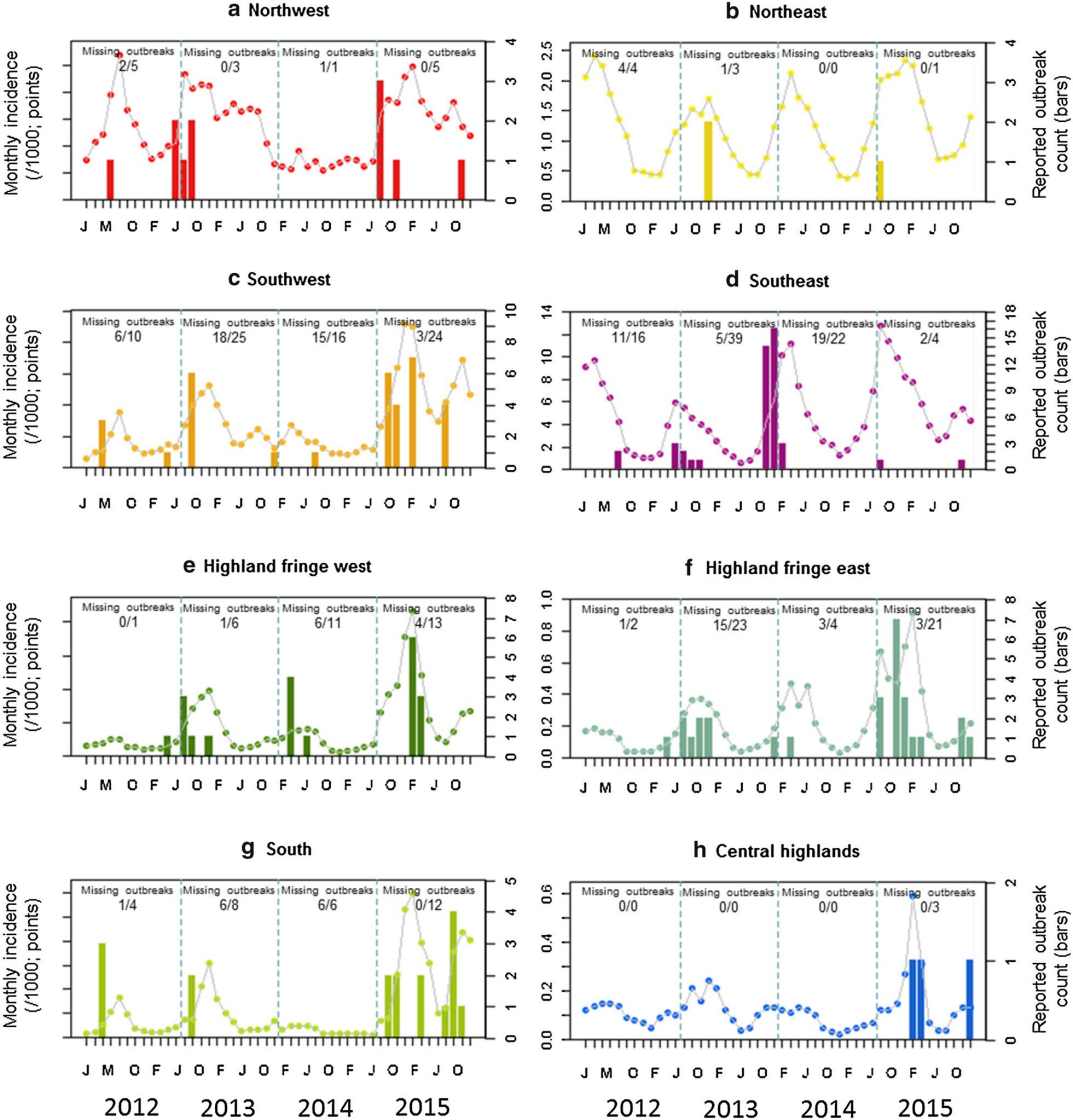
Temporal correspondence between reported malaria case incidence (*left-hand y axis*; plotted as points) and outbreak reports (*right hand y axis*; plotted as *barplots*) aggregated by ecozone for 2012–2015. Of the 292 outbreaks reports, nearly half (n = 133) provided no supporting information about the affected month so these could not be included in the monthly outbreak plots. The total annual outbreaks reported per ecozone is specified in the *plots* (fraction denominator), with the numerator summarizing the number of outbreaks missing from the *bar plots* due to incomplete information

The occurrence of outbreaks in relation to the overall burden of malaria is considered in Fig. 7. Records of the outbreaks were incomplete, with 133 of the 292 outbreak reports in the database not specifying the affected month. The outbreaks barplots in Fig. 7 are therefore incomplete, but nevertheless suggest a relationship with the intensity of background transmission. Lower endemicity areas, such as the two Highland fringe ecozones, have peaks in incidence corresponding with months when outbreaks were reported; while higher incidence areas, such as along the East coast and the Northwest ecozone, trends in malaria incidence do not correspond to outbreak periods. For example, in the Southeast, the year with fewest reported outbreaks (2015; n = 4) was when incidence was greatest.

## Discussion

An updated sub-national stratification of Madagascar is proposed which reflects contemporary transmission intensity. Within these limits, the epidemiology of malaria is described as evidenced by routinely reported health facility data made available to the NMCP. From this evidence base, the National Programme formulates control policies, oversees epidemiological monitoring, and assesses policy impacts. The eight ecozones may not necessarily each require bespoke control schemes, but these allow greater sub-national epidemiological insight, and therefore flexibility in targeting of interventions based on local needs. These sub-divisions are not static, but instead must be periodically updated to reflect shifts in the current patterns of transmission, though with a frequency that does not prevent temporal trends being assessed in the short term. The narrow time window presented here is the product of decades of malaria control, so the epidemiology described cannot be interpreted as representing the fundamental niche of malaria in Madagascar.

Malaria is a disease of increasing public health concern in Madagascar. Since 2010, the number of reported RDT-confirmed cases has quadrupled across the country. When adjusted for numbers of tests performed (thus adjusting for (i) reporting rates, (ii) the unavailability of diagnostics, and (iii) the scale-up of access to diagnostic testing through community health worker programmes [42]), the RDT positivity rate increased by 50 % between 2010 and 2015, reaching 50 % slide positivity in 2015. Despite this evidence of recent resurgences, the incidence of malaria in Madagascar remains among the lowest of the sub-Saharan African region, with high levels of intervention coverage, particularly of LLINs [8, 14]. This juncture therefore represents an important opportunity to re-evaluate strategies and reverse the recent negative trends. For instance, while LLIN coverage may be high, treatment-seeking and adherence to recommended artemisinin combination therapy (ACT) policies appears weak [8, 43].

Although the majority of the malaria burden remains in the Southeast (42 % in 2015), malaria has increased across all areas in year-round significance, increasing in intensity during both high (December–April) and low transmission periods. The Central highlands seem to be the exception, but the epidemiology differed there, consisting primarily of imported cases (NMCP, pers. comm.), and with very low incidence compared to other ecozones. Outbreaks, defined in this context as unexpectedly high or rapid increases in transmission or evidence of autochthonous transmission in areas considered at low risk, appear to associate closely with overall burden trends in lower-transmission ecozones. A more detailed view of changes in case numbers at health facilities affected by outbreaks would allow more robust insights into the relative contribution of outbreak-driven transmission to overall caseloads across the country.

An important missing piece from this study’s narrative is the relative contribution of each *Plasmodium* species to the regional case burden. This detail is not recorded in the data. Prevalence surveys indicate a predominance of *P. falciparum* across most areas, but regional pockets of *Plasmodium vivax* and sporadic cases of *Plasmodium ovale* and *Plasmodium malariae* are also detected [24, 25, 29, 44, 45]. A longitudinal dataset on the contribution of each species to morbidity from routine reporting would improve the evidence-base for determining the value of introducing non-*P. falciparum*-specific intervention policies.

The recent trends described in this study stand in stark contrast to the country’s stated ambitions to start the transition towards malaria elimination. The country’s current National Strategic Plan (2013–2017) aims for pre-elimination status by end 2017, with zero malaria deaths and the reduction of test positivity rates to <5 % in 15 % of districts, and the halving of the positivity rate in the remaining districts relative to 2013 baseline data [21, 39]. Overall, however, 2013–2015 saw a 34 % increase in the positivity rate. Acknowledgement of these trends by the NMCP led to a revision of the National Strategic Plan in 2015, with adjustment of their initial goals now more aligned with achieving burden control than elimination [21]. The drive to control and eventually eliminate malaria from Madagascar receives strong financial backing from the international community, with disbursements in excess of $400 million since 2004 when collaborative efforts were galvanized and intensified [8, 11, 46]. In 2006–2010, resources for malaria control in Madagascar corresponded to higher than average per-capita funding across the African region [8, 47]. Since this time though, the country’s political crisis has severely impacted on the country’s socio-economic status and associated health system infrastructure and staffing, impeded external funding to the country thus preventing intervention roll-out (see the Global Fund disbursement delays in Fig. 1), and complicated the control programme management by preventing direct bilateral relationships between the MoH and donors [21].

The situation in Madagascar therefore reflects what has been widely reported from other areas, that withdrawal of core funding is closely associated with rapid and predictable resurgence of malaria [48, 49] (Fig. 1). Despite high coverage rates of interventions around 2013, interrupted funding led to delays in sustaining these levels. Periods of limited funding are when reliable epidemiological data become all the more important to inform efficient planning and optimize resource allocation. Such data require robust processing at all stages of the chain from the probability of patients seeking treatment at a health facility, through diagnosis, through data reporting to data management and evaluation at the central level. More than half the Malagasy population live further than 5 km (roughly corresponding to an hour’s walk) from their nearest health facility. This likely contributes substantially to the treatment-seeking rate that ranged in 2013 from 21.7 to 49.1 % across different parts of the country [24, 43, 50, 51], with many patients suffering fevers resorting to self-medication instead (including both pharmaceutical and traditional herbal remedies) [52]. Therefore, most cases of fever will never interact with the country’s formal health system. The routine HMIS had a largely consistent reporting rate from health facilities across 2010–2014 at approximately 60 %, though rising to 75 % in 2015 (Fig. 4a), meaning that almost half of health centre reports were not recorded in the NMCP’s databases for most of the time period reviewed. Further reinforcement of the incompleteness of the datasets is the disparity between distributed diagnostic kits and the test results reported (Fig. 4b). The received outbreak surveillance data are also incomplete, evidenced by nearly half of reports not being month-specific (Fig. 7).

Madagascar has a well-documented history of devastating epidemics, with the earliest reported outbreak around 1887 coinciding with the establishment of rice farming [21]. Outbreaks both in Madagascar and globally have primarily occurred in the context of socio-economic and political upheavals [48] when there has been a breakdown of the control programme structure, including surveillance and reporting channels. Implicit in the programmatic concept of an outbreak is that the event triggers an emergency response from the responsible public health institution to curtail the exacerbation of the situation. An efficient surveillance system will allow outbreaks to be anticipated and intervention resources to be mobilized ahead of transmission developing out of control.

The reporting system in Madagascar was described in 2015 by the US President’s Malaria Initiative as “extremely weak”, hindered in part by a critical shortage of health staff at all levels, with human resources unevenly distributed across the country [11]. To further investigate this, a nationally representative review of the health reporting system was commissioned in 2015 to qualitatively and quantitatively assess the system’s functionality (thus supplanting the need for proxy indictors of completeness). This included detailed interviews with staff at all levels of the reporting pyramid, alongside comparisons of raw data to assess the completeness and accuracy of reporting from doctors’ records up to the central database. Preliminary results suggest that both the quantity and quality of reported health facility data were weak. Reporting timeliness was particularly affected, with only 8 of 45 districts reviewed in January 2015 reporting within the MoH’s defined schedule [31]. Reporting completeness varied across the country, being highest in the central highlands, and lowest in the tropical coastal areas and desert south. A further concern identified by the surveys related to the lack of health professionals to make use of the data and identify trends specific to their local catchment population: interpretation of the data was principally limited to the central level, and not performed in situ. The investigators’ perspectives of the NMCP’s data in particular, was that the existence of a database was a strength of their epidemiological monitoring unit. Weaknesses of the data included the complexity of the reporting system, its fragmented nature and the difficulty of assimilating the data for analysis, logic checks are not routinely performed on the data, and confusion existed regarding some of the calculations used to derive the metrics reported [31]. Surveys actively assessing stocks of diagnostic kits in health centres suggest that the passively reported data on stock-out events that were available for review here (Fig. 4c) underestimate the true incidence of stock-out events [49]. This indicates that the true rate of under-diagnosis due to diagnostic stock-outs and knock-on impact on reported case numbers may be even greater than suggested. Stakeholders are aware of these limitations to the reporting system, and a concerted effort to address them is ongoing.

Given the need for annual case estimates to assess both national- and global-level trends in malaria incidence and to monitor progress towards pre-defined milestones, the World Health Organization’s (WHO) Global Malaria Programme and collaborators have developed methods to account for weaknesses in routine HMIS data [8, 14, 53, 54]. Two main approaches exist to estimate malaria incidence [53], each best suited to different countries according to their programme control phase and the strength of their disease surveillance system. Method 1 is favoured by the WHO for countries outside Africa, where routine reporting is considered more reliable. This method uses parameters to adjust MoH routine HMIS data to account for (i) incomplete case detection by health facilities; (ii) the likelihood of over-diagnosis of malaria among patients with fevers; and, (iii) public/private treatment-seeking preferences [53, 55]. Method 2 uses a cartographic approach based on population prevalence surveys to develop spatial and temporal models of the incidence of clinical disease; this has been the main approach followed by MAP to derive global endemicity maps of *P. falciparum* and *P. vivax* transmission [14, 56, 57], and is used by the WHO to estimate case numbers from most sub-Saharan African countries where routine reporting is considered too weak for Method 1 estimation. A subset of low-transmission African countries is also considered to have sufficiently strong routine reporting data to allow support for Method 1-based estimates. The implication being that these countries have a sufficiently functional health system to have robust surveillance and reporting systems in place within the context of a functional and effective malaria control programme. The sub-Saharan African countries included in this category are Botswana, Cabo Verde, Eritrea, Namibia, South Africa, Swaziland, and Zimbabwe (all of which are classified as being “on track for >75 % decrease in incidence 2000–2015”), and Madagascar (“less than 50 % change in incidence projected 2000–2015”) [8]. Nevertheless, the WHO lists Madagascar among the eight sub-Saharan African low-transmission countries with sufficiently accurate HMIS data to permit convincing estimates to be made from simple adjustment of the numbers of reported cases. This study explores the routine HMIS data available to the Malagasy NMCP which underpins this perhaps contradictory classification. Of the subset of African Method 1 countries, Madagascar had the highest endemicity in 2015 [8, 14] and among the lowest access to the formal health system [43]. The magnitude of the uncertainty in the reported case numbers is evident by the WHO’s case estimate for Madagascar in 2013. Although 387,045 cases were reported by Madagascar to the World Malaria Report for 2013, once adjusted using Method 1, the estimated case number for 2013 was 1.2 million (0.75–2.10 million) [8], therefore corresponding to more than three times the reported RDT-confirmed numbers. Given these acknowledged uncertainties and the recent resurgence of transmission with variable sub-national patterns, a re-consideration of the method applied to case estimation in Madagascar would be worthwhile. Regular nationally representative Malaria Indicator Surveys and other prevalence surveys take place, which provide a rich source of malariometric data complimentary to the HMIS data.

If Madagascar is to remain within the Method 1 countries and have its case estimates derived from adjustment of the routine reporting data, the spatial heterogeneity of transmission characteristics and of reporting robustness discussed in this paper would be a valuable addition to the methodology instead of relying on a single set of parameters. The data adjustment parameters show widespread variation between ecozones, with treatment-seeking rates in the Fringe areas less than half that of the South (21.7 and 49.1 %, respectively) [24]. Similarly, health facility reporting rates differ between areas, and, although no correlation could be identified from the aggregated district-level data reviewed here, it is likely that some association between health centre isolation and reporting quality and timeliness exists. No single set of national-level summary adjustment parameters, as per the current WHO Method 1 protocol, could capture this heterogeneity. The sub-national variation in transmission intensity is reflected by factors such as age-specific incidence patterns (Fig. 3b). The Central highlands ecozone, where transmission is lowest, has no significant age-specific patterns with the whole population at similar risk of infection. In higher endemic areas, younger children suffer the highest burden of disease, likely due to the development of immunity in older age groups from repeated exposure in childhood. To adequately adjust health facility data from the HMIS, this spatial variability ought to be taken into account. In parallel to this, large numbers of prevalence surveys have been performed across the country as part of three completed MIS surveys from 2011, 2013, and April 2016 (results expected end 2016), among others [29]. These community-based surveys have shown the same increase in prevalence as the HMIS health-facility based case data. While these prevalence surveys are generally better standardized than HMIS data, they are much less comprehensive in space and time. Comparison of the estimates from these two independent methods (routine data-based and cartographic) and datasets would be valuable.

## Conclusion

Madagascar is highly diverse, not least in terms of its malarial epidemiology, and accordingly requires spatially specific control planning. This paper presents an overview of the last 5 years of HMIS data, the period immediately following a political crisis and the nation’s subsequent exclusion from the international community until May 2014. Associated with these events, malaria control efforts have been hindered, resulting in rapid resurgences of transmission [49], with ecozones previously considered to be low transmission zones, such as the Southwest and South, now becoming stable endemic areas, reflected by differential patterns in age-specific incidence, for example.

No single approach for malaria control can be applied across the island, or even binary high/low transmission approach given the peculiarities of transmission in different ecozones. Regular epidemiological assessments are therefore recommended at the ecozone levels. The NMCP is frequently restricted to being reactive rather than proactive and unable to anticipate demands (e.g., in terms of anticipating commodity needs or anticipating outbreaks). Closer analysis of data trends in real-time, would be valuable to support the NMCP. For instance, it will be important to closely evaluate the 2016 data to assess the impact of the 2015–2016 LLIN distribution programme, and determine how rapidly the sharp increase of cases in 2015 can be reversed to shift the country in the direction of its elimination goals.

This study comes at the end of the Millennium Development Goal era (2000–2015), which aimed to have “halted by 2015 and begun to reverse the incidence of malaria” (Target 6.C) [12]. In Madagascar this was clearly being achieved from 2000 to 2009, though the reverse has occurred since 2010 (Figs. 1, 2, 3). Using the year 2000 as a baseline descriptor may not be a very pertinent approach for evaluating the current status of progress in malaria control. The situation in 2000 was one of essentially no financial or political commitment to large-scale intervention roll-out. Most reported cases would have been presumptively diagnosed based on symptoms (likely over-estimating case numbers), given that rapid field-based diagnostics were only introduced in 2007. More pertinent than noting a decrease in caseload since 2000 is the concerning increase in burden since 2010 when funding sources were substantially increased and malaria control was widespread across Madagascar.

While it is important to interpret the imperfect health facility data with caution [42, 58], the available data do provide detailed insight into the relative differences in epidemiology sub-nationally. However, given that between a quarter and half of health facility reports are missing from the NMCP’s database, the absolute number of cases reported is evidently a major underestimate. Approaches to quantify estimates of the absolute annual burden would be useful in attempting to deduce a more robust estimate of overall burden rather than relying on an incomplete database as is currently the case by the NMCP. Nevertheless, the HMIS data present a convincing case for the growing challenge of malaria across all areas of Madagascar.

## Additional files

**Additional file 1.** Summary of data sources consulted. The table provides details of the data sources used for each figure in the paper.

**Additional file 2.** Malaria Atlas Project (MAP) modelled maps of *Plasmodium falciparum* parasite prevalence (*Pf*PR) in 2010–2015. This document provides additional information about the modelled prevalence map that was used to stratify Madagascar into contemporary ecozones.

**Additional file 3.** Tabulation of regions and districts by ecozone. Administrative regions and districts of Madagascar classified according to the newly designed ecozones.

**Additional file 4.** Additional plots of the trends of reported malaria case data in Madagascar (2010–2015). This file provides additional analysis of the temporal trends in the reported malaria data. Data are adjusted for reporting and diagnostic shortages, and trends are examined using autocorrelation plots, generalized additive models and linear regression models.
