# Supplementary material for: Contemporary epidemiological overview of malaria in Madagascar: operational utility of reported routine case data for malaria control planning
Source: Malar J. 2016 Oct 18;15:502. doi: 10.1186/s12936-016-1556-3 (PMC5070222; doi:10.1186/s12936-016-1556-3)
Supplement: Supplementary file 1 — Additional file 1. Summary of data sources consulted. The table provides details of the data sources used for each figure in the paper. [file 12936_2016_1556_MOESM1_ESM.pdf]

*Supplementary table: Summary of data sources*

| Manuscript figure | Dataset | Data type | Source | Method used to generate data | Time period |
|---|---|---|---|---|---|
| Figure 2C | *Plasmodium falciparum* endemicity (*Pf*PR) | Raster | Malaria Atlas Project (www.map.ox.ac.uk) | Geostatistical modelling | 2010-2015 |
| Figure 2E | Digital Elevation | Raster | Shuttle Radar Topography Mission (SRTM) | Remote sensing | n.a. |
| Figure 2F | Rainfall (mean annual total) | Raster | NASA Tropical Rainfall Measuring Mission (TRMM) | Remote sensing | 2010-2014 |
| Figure 2G | Temperature suitability | Raster | Malaria Atlas Project; Weiss *et al* 2015 | Remote sensing and modelling | n.a. |
| Figure 2H | Land-cover use | Raster | IGBP MODIS annual landcover product MCD12Q1 | Remote sensing | ca. 2015 |
| Figure 3A (case data) | Reported RDT+ cases | Case data | NMCP, Madagascar | Routine health metric reporting system | 2010-2015 |
| Figure 3B (population data) | Case incidence | Census data | Ministry of Health, Madagascar | Population census (1993) adjusted to fixed growth rate | 2010-2015 |
| Figure 4A | Count of health facility reports | Health facility reports | NMCP, Madagascar | Routine health system reporting | 2010-2015 |
| Figure 4B | RDT result reporting rate | Health facility reports | NMCP, Madagascar | Routine health system reporting | 2014 |
| Figure 4C | RDT stock-out incidence | Health facility reports | NMCP, Madagascar | Routine health system reporting | 2012-2014 |
| Figure 5A | Population density map | Raster | WorldPop (www.worldpop.org) | Geostatistical modelling adjusted to UN population estimates | 2015 |
| Figure 5B | Health centre accessibility to population | Health facility reports | NMCP, Madagascar | Health facility reporting | ca. 2015 |
| Figure 5C | Health centre accessibility to health district offices | Health facility reports | NMCP, Madagascar | Health facility reporting | ca. 2015 |
| Figure 6 | Documented outbreaks | Health facility reports | NMCP, Madagascar | Integrated Diseases Surveillance and Response System | 2012-2015 |
| Figure 7 | *Superimposes Figure 3B with Figure 6* | | | | |
| Additional file 2 | *Plasmodium falciparum* endemicity (*Pf*PR) | Raster | Malaria Atlas Project (www.map.ox.ac.uk) | Geostatistical modelling | 2010-2015 |
| Additional file 4 | Reported RDT+ cases | Case data | NMCP, Madagascar | Routine health metric reporting system | 2010-2015 |
