# Supplementary material for: Contemporary epidemiological overview of malaria in Madagascar: operational utility of reported routine case data for malaria control planning
Source: Malar J. 2016 Oct 18;15:502. doi: 10.1186/s12936-016-1556-3 (PMC5070222; doi:10.1186/s12936-016-1556-3)
Supplement: Supplementary file 2 — Additional file 2. Malaria Atlas Project (MAP) modelled maps of Plasmodium falciparum parasite prevalence (PfPR) in 2010–2015. This document provides additional information about the modelled prevalence map that was used to stratify Madagascar into contemporary ecozones. [file 12936_2016_1556_MOESM2_ESM.docx]

**Additional file 2: Malaria Atlas Project (MAP) modelled maps of *Plasmodium falciparum* parasite prevalence (*Pf*PR) in 2010-2015.**

This Supplementary file provides background information to the endemicity maps that were used to determine the sub-national stratifications of malaria transmission intensity.

The Malaria Atlas Project (MAP) uses geostatistical modelling to generate maps of malaria endemicity (quantified as the prevalence rate “PR” of *Plasmodium* infection across a community at a given point in time). The models generate predictions which are continuous in space and in time, with outputs summarised to 5x5km pixel grid with monthly temporal resolution. The outputs used here are mean summary maps. Associated uncertainty metrics were not accounted for in this paper’s zonal stratification, only the mean maps.

The models are informed by a range of inputs, including measures of malaria transmission intensity (both annual case incidence data and cross-sectional community surveys of parasite prevalence) [1, 2], environmental covariates [3], and coverage rates of key control interventions [4]. The underlying evidence-base datasets, together with the annual mean maps, are freely available from the MAP website ([www.map.ox.ac.uk](http://www.map.ox.ac.uk)) for all African countries across the 2000-2015 time window. The original publication of this work provides full details of the geostatistical model developed for mapping [2].

Maps summarising the predicted annual prevalence of *Plasmodium falciparum* (*Pf*PR) infection (mean of the monthly predictions) across the 2010-2015 time period are shown in Figure S1.1. Figure S1.2 is the mean of these annual maps, and was used to guide the subnational stratification of malaria transmission shown in Figure 2D.

**
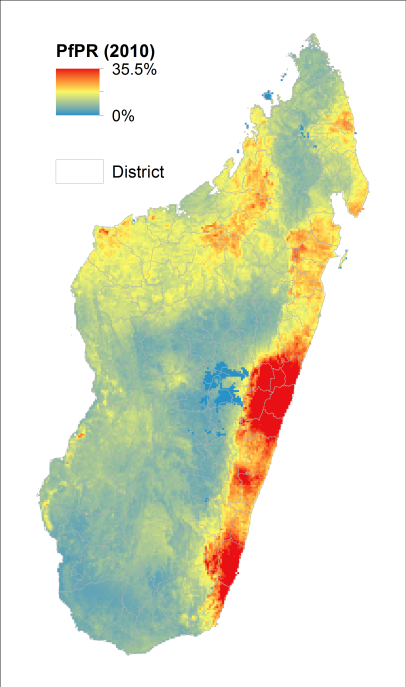

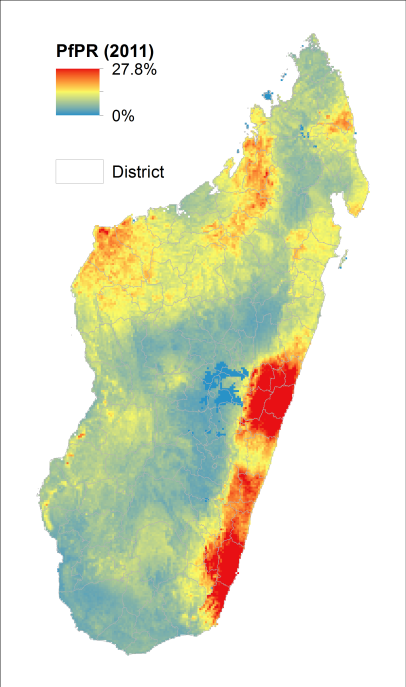

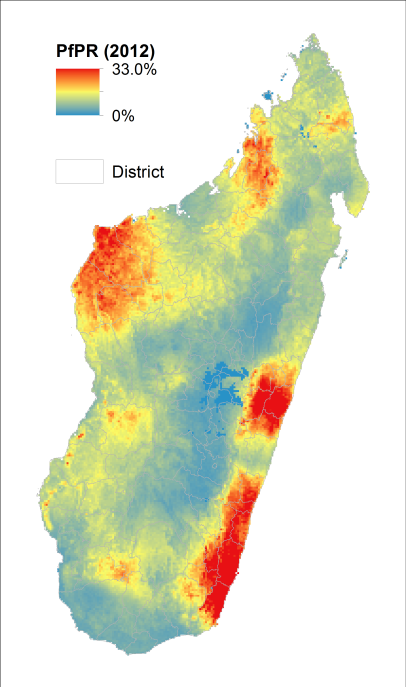

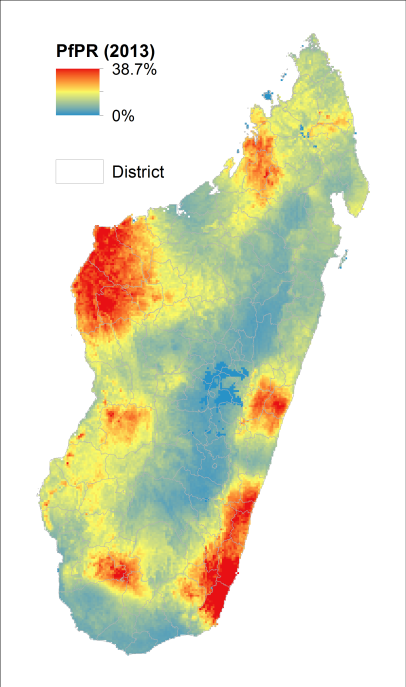

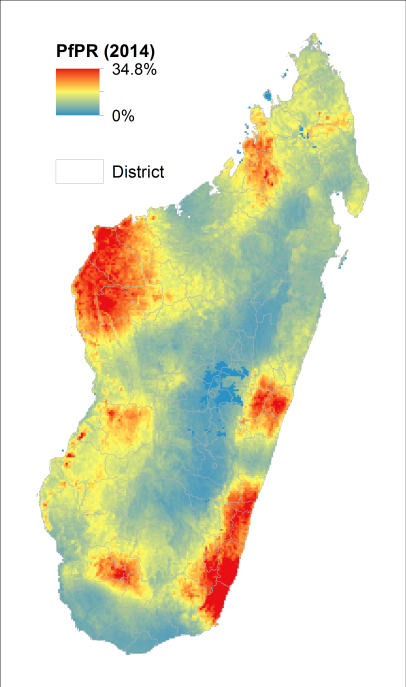

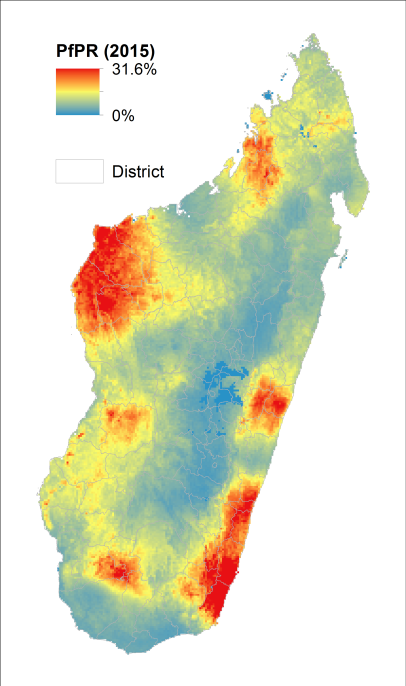
**

**Figure S1.1. Modelled annual *Pf*PR estimates**, plotted at 5x5km resolution. Source: Malaria Atlas Project (MAP; [www.map.ox.ac.uk](http://www.map.ox.ac.uk); [2]).

**
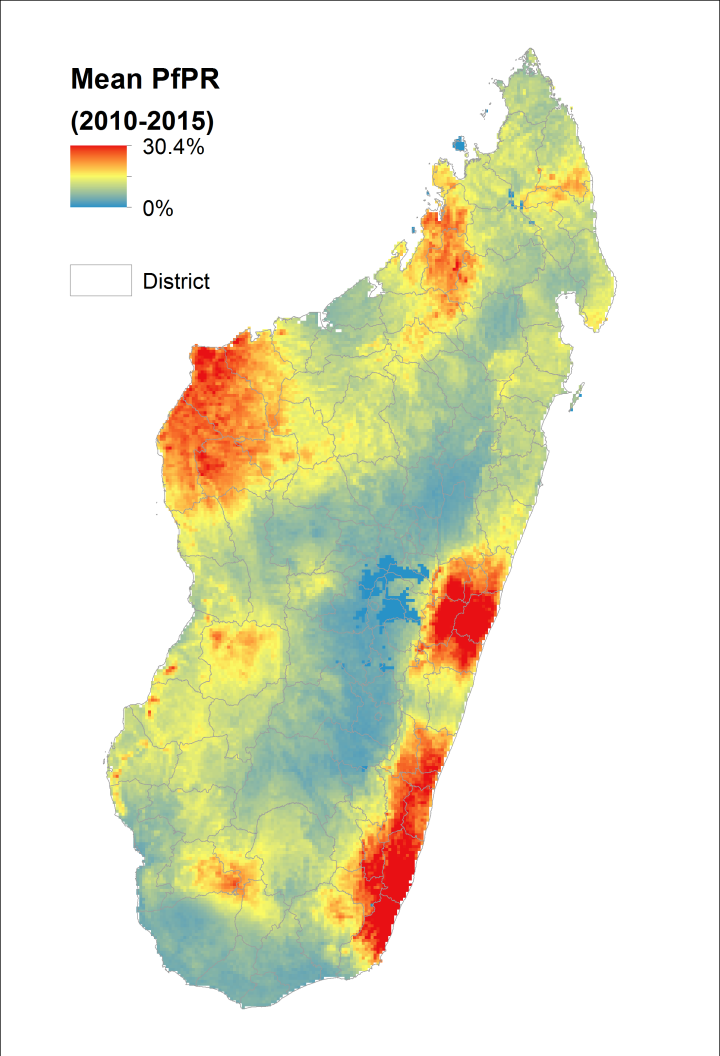
**

**Figure S1.2. Summary map of *Pf*PR 2010-2015** (as plotted in Figure S1.1).

**References**

1. Guerra CA, Hay SI, Lucioparedes LS, Gikandi PW, Tatem AJ, Noor AM, Snow RW: **Assembling a global database of malaria parasite prevalence for the Malaria Atlas Project.** *Malar J* 2007, **6:**17.

2. Bhatt S, Weiss DJ, Cameron E, Bisanzio D, Mappin B, Dalrymple U, Battle KE, Moyes CL, Henry A, Eckhoff PA, et al: **The effect of malaria control on *Plasmodium falciparum* in Africa between 2000 and 2015.** *Nature* 2015, **526:**207-211.

3. Weiss DJ, Mappin B, Dalrymple U, Bhatt S, Cameron E, Hay SI, Gething PW: **Re-examining environmental correlates of *Plasmodium falciparum* malaria endemicity: a data-intensive variable selection approach.** *Malar J* 2015, **14:**68.

4. Bhatt S, Weiss DJ, Mappin B, Dalrymple U, Cameron E, Bisanzio D, Smith DL, Moyes CL, Tatem AJ, Lynch M, et al: **Coverage and system efficiencies of insecticide-treated nets in Africa from 2000 to 2017.** *Elife* 2015, **10.7554/eLife.09672**.
