# Supplementary material for: Contemporary epidemiological overview of malaria in Madagascar: operational utility of reported routine case data for malaria control planning
Source: Malar J. 2016 Oct 18;15:502. doi: 10.1186/s12936-016-1556-3 (PMC5070222; doi:10.1186/s12936-016-1556-3)
Supplement: Supplementary file 4 — Additional file 4. Additional plots of the trends of reported malaria case data in Madagascar (2010–2015). This file provides additional analysis of the temporal trends in the reported malaria data. Data are adjusted for reporting and diagnostic shortages, and trends are examined using autocorrelation plots, generalized additive models and linear regression models. [file 12936_2016_1556_MOESM4_ESM.docx]

**Additional file 4: additional plots of the trends of reported malaria case data in Madagascar (2010-2015).**

This Additional file provides additional analysis of the temporal trends in the reported malaria data. As per the main paper, analyses are conducted at the ecozone level, and colour coding is consistent with the ecozones as mapped in Figure 2D (reproduced below).

The datasets are considered here in sequence reflecting the Results section of the main manuscript. First, additional results about the raw reported case data are given. Second, these figures are adjusted to population counts to consider incidence to allow comparisons between very differently sized and populated spatial areas, as well as enabling assessments of temporal trends. Third, to account for potential limitations from incomplete reporting, malaria case reports are adjusted to the total reported consultations. Fourth, to account for potential stock-outs of diagnostic tests, trends in the test positivity rate are reviewed.

Autocorrelation plots are used to illustrate the temporal structure of the time series, including any seasonality component. The plots compute the strength of the correlation between consecutive points in the time series. Each time lag (x axis) corresponds to a month, and the autocorrelation metric indicates the relative strength of correlation across increasing numbers of time lags. Time lag 0 has a reference autocorrelation of 1, indicating that all values are 100% correlated with themselves. Time lag 1 indicates the relative strength of correlation between time points 1 month apart, time lag 2 is correlation between points 2 months apart etc. Autocorrelation is considered present if above (positive) or below (negative) the horizontal dashed blue lines (p<0.05).

Generalized additive models were applied to time series where visual temporal trends were hard to discern from the stochasticity across the time points. These models smooth the trends allowing visual comparisons across years and between ecozones. Linear regression models were used to look at long-term trends across years and test the statistical significance of temporal changes across the overall trends.

This Additional file aims to provide a comprehensive overview of the intra- and inter-ecozone temporal trends. As in the rest of the paper, the data examined here all comes from the Madagascar National Malaria Control Programme databases. For consistency, the colour scheme applied to the plots in this Supplementary file is consistent with that used in the main manuscript. Figure 3D is reproduced here for reference.


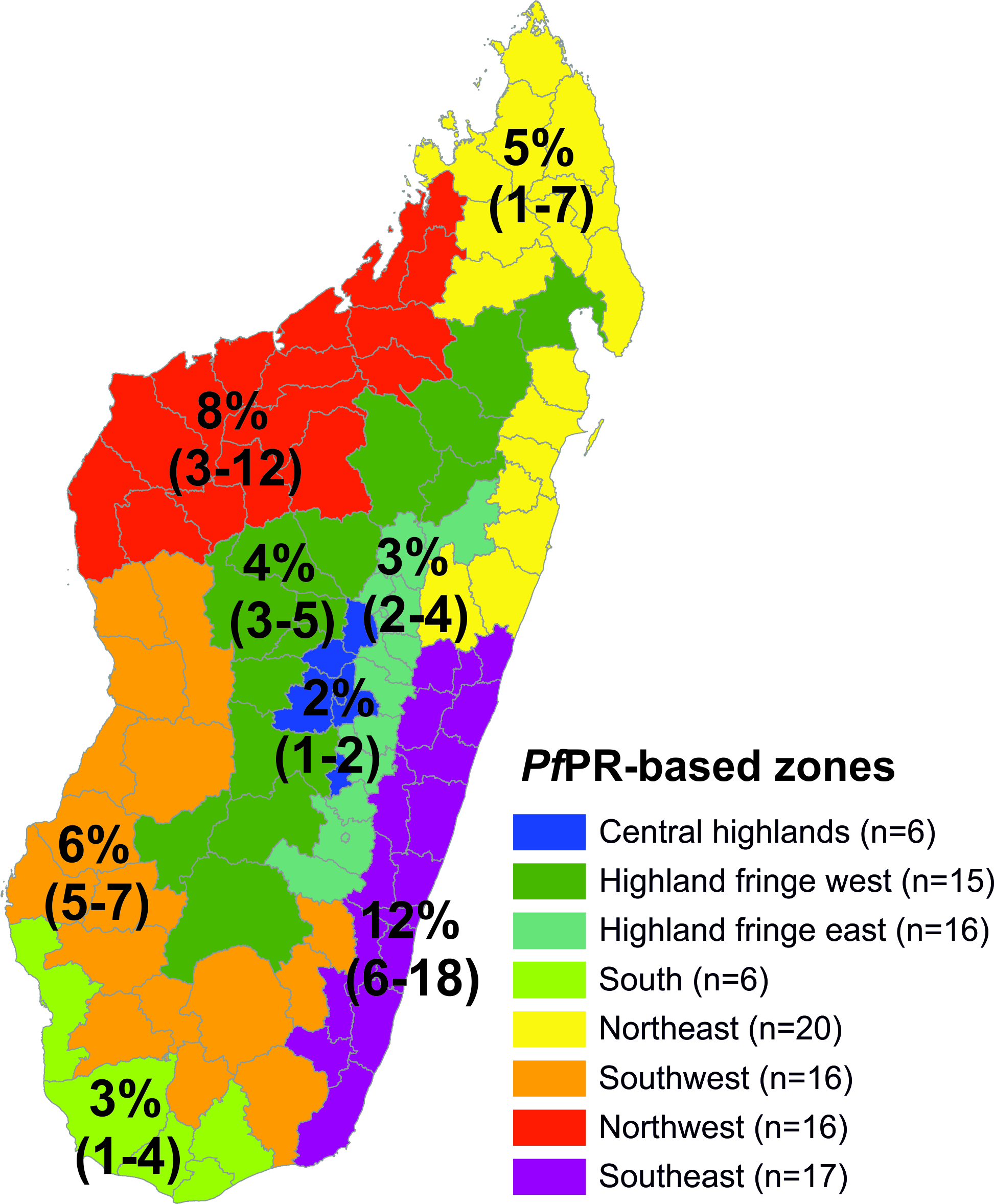


**Figure 3D: updated malaria transmission ecozone stratification.** Regional figures correspond to the mean district-level *Pf*PR value (2010-2015) and the min-max *Pf*PR values from the districts in each ecozone.

*Figure reproduced from the main manuscript.*

The figures and tables included in this Additional file are:

1. **Reported RDT+ case data** (supplementary plots to Figure 3A).

Figure S2.1. Disaggregated plots showing total reported RDT+ cases in each ecozone.

1. **Reported RDT+ case incidence** (supplementary plots to Figure 3B).

Figure S2.2. Autocorrelation plots of the all-age monthly incidence of RDT-confirmed cases, analysed by ecozone.

Figure S2.3. Linear model of the national-level temporal trends of malaria incidence 2010-2015.

Figure S2.4.A. Linear models of all-age monthly incidence, by ecozone.

Figure S2.4.B. Summary plot of the linear models from each ecozone.

Table S2.1. Summary statistics of the linear models of each ecozone’s time series of case incidence (2010-2015).

1. **Reported contribution of confirmed malaria cases to overall health facility consultations** (supplementary plots to the “Trends in routine malaria case reports” section of the Results).

Figure S2.5. Proportion of all consultations reported monthly from each ecozone resulting in an RDT+ diagnosis.

Figure S2.6. Linear models of the time series of the proportion of RDT+ consultations (2010-2015).

Figure S2.7. National-level linear regression model of the proportion of health facility consultations that were RDT+ over the 2010-2015 time series

Table S2.2. Summary statistics of the linear models of each ecozone’s time series of the proportion of health-centre consultations that were RDT+ during the 2010-2015 period.

1. **Reported proportion of health facility consultations resulting in an RDT+ diagnosis** (supplementary plots to the “Trends in routine malaria case reports” section of the Results).

Figure S2.8. Test positivity rates by ecozone (2010-2015).

Figure S2.9. Autocorrelation in the time series of test positivity rates across the 2010-2015 time period.

Figure S2.10. Generalized additive models of the change in RDT positivity rate over time by ecozone.

Figure S2.11A. Linear regression models of the change in RDT positivity rate over time by ecozone.

Figure S2.11B. Linear regression model of the time series of national-level test positivity rate.

Table S2.3. Summary statistics of the linear models of temporal trends in diagnostic test positivity rates (2010-2015).

1. **Reported RDT+ case data** (supplementary plots to Figure 3A).


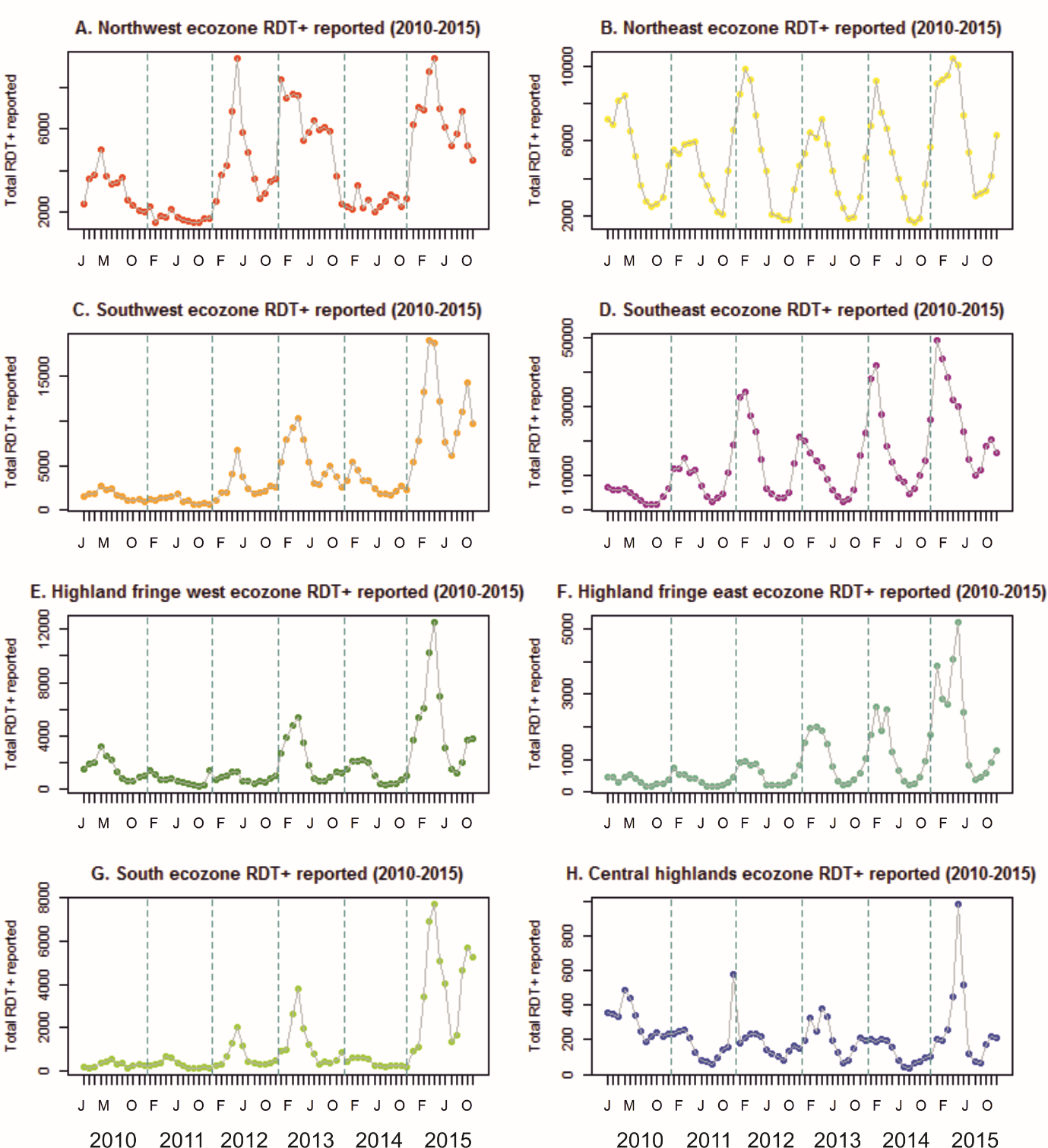


**Figure S2.1. Disaggregated plots showing total reported RDT+ cases in each ecozone.** Y-axis scaling is adjusted to each ecozone.

1. **Reported RDT+ case incidence** (supplementary plots to Figure 3B).

Temporal trends in the incidence of RDT-confirmed cases were illustrated using autocorrelation plots to see the relative degree of correlation across the time series (Figure S2.2). Temporal patterns indicating repeating annual patterns show autocorrelation around the 12 month lag. These are most visible from the eastern areas (Northeast, Southeast, Highland fringe east). Other areas did not show such strong correlations in incidence between years.

Assessment of changes over time to the incidence of malaria (based on reported cases) was investigated at the national (Figure S2.3) and ecozone level (Figure S2.4).


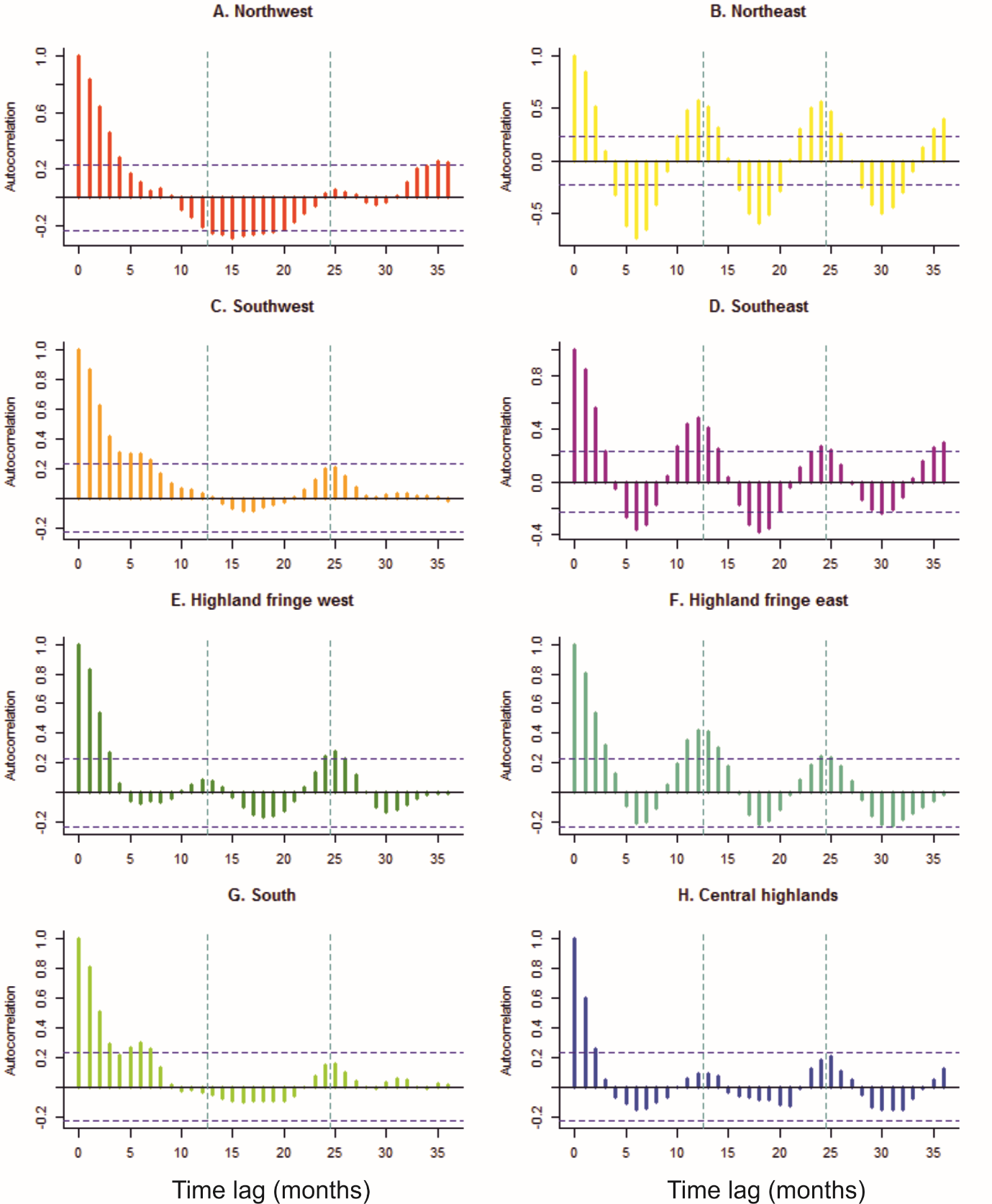


**Figure S2.2. Autocorrelation plots of the all-age monthly incidence of RDT-confirmed cases, analysed by ecozone.** Vertical bars plot the influence of neighbouring time points on each month’s values relative to 1 at time lag 0 (i.e. values are 100% influenced by themselves). Bars within the horizontal dashed bracket of the plots indicate no autocorrelation. Positive autocorrelation points indicate a positive correlation; and vice versa for negative autocorrelation values. Dotted lines represent p<0.05 statistical significance.


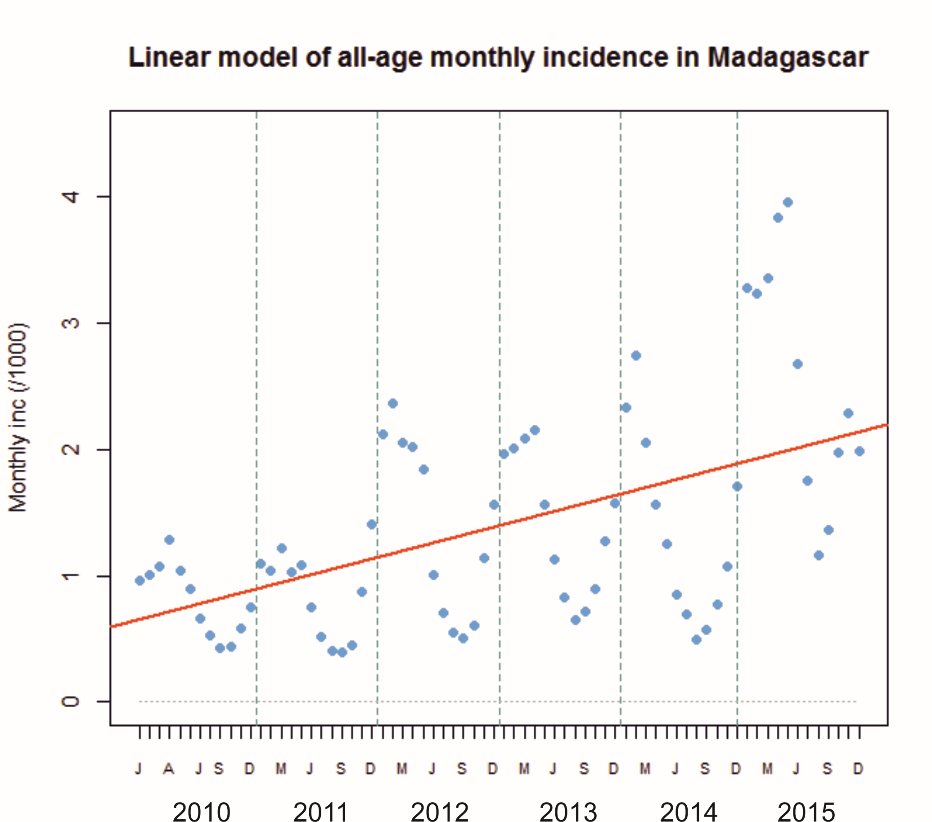


**Figure S2.3. Linear model of the national-level temporal trends of malaria incidence 2010-2015**. The model (red line) indicates a highly significant increasing trend in incidence across the time period, with a monthly effect size (β) of 0.020924 (P <0.00001).


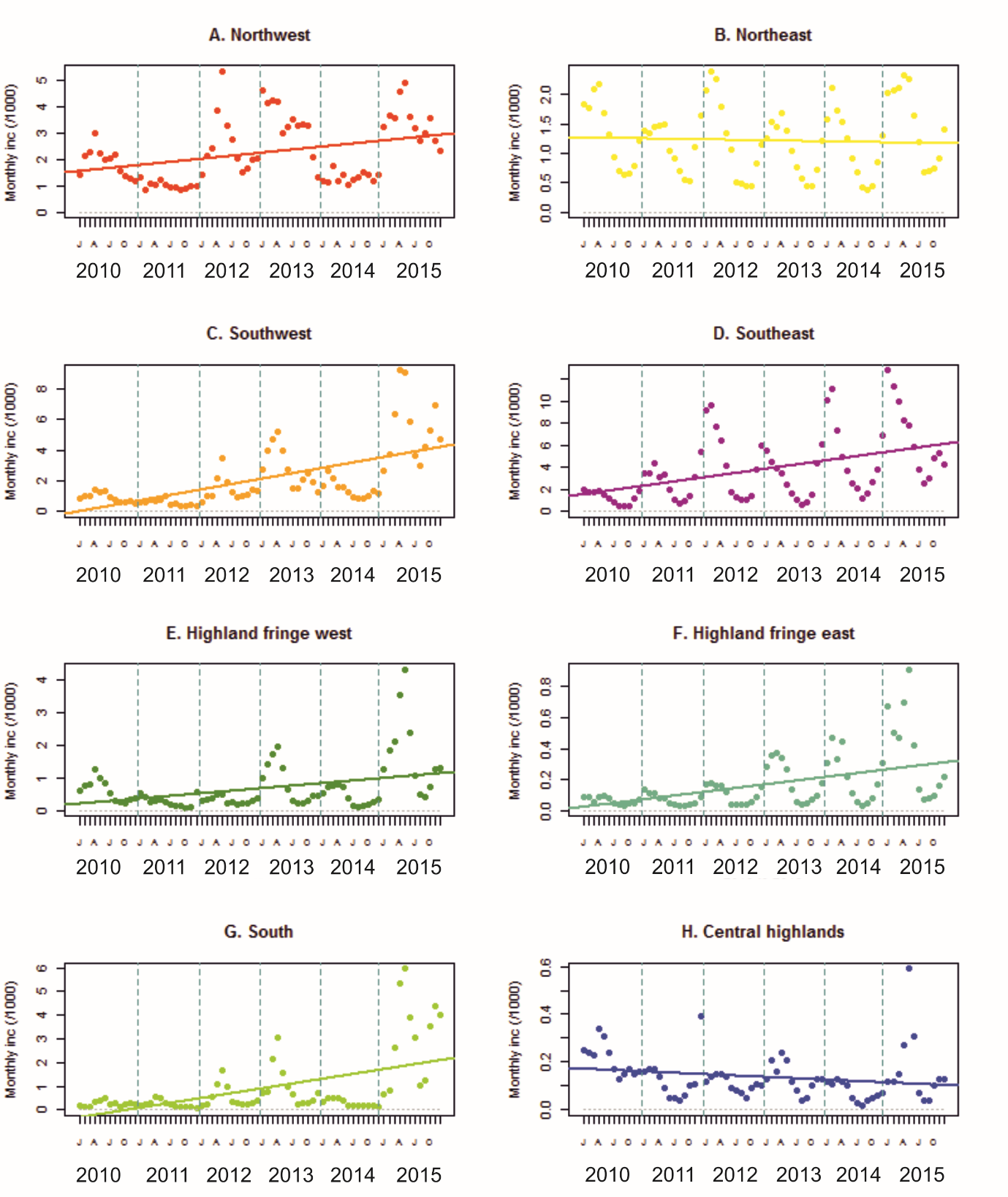


**Figure S2.4.A. Linear models of all-age monthly incidence, by ecozone**. Plots show the underlying time series dataset informing the models.


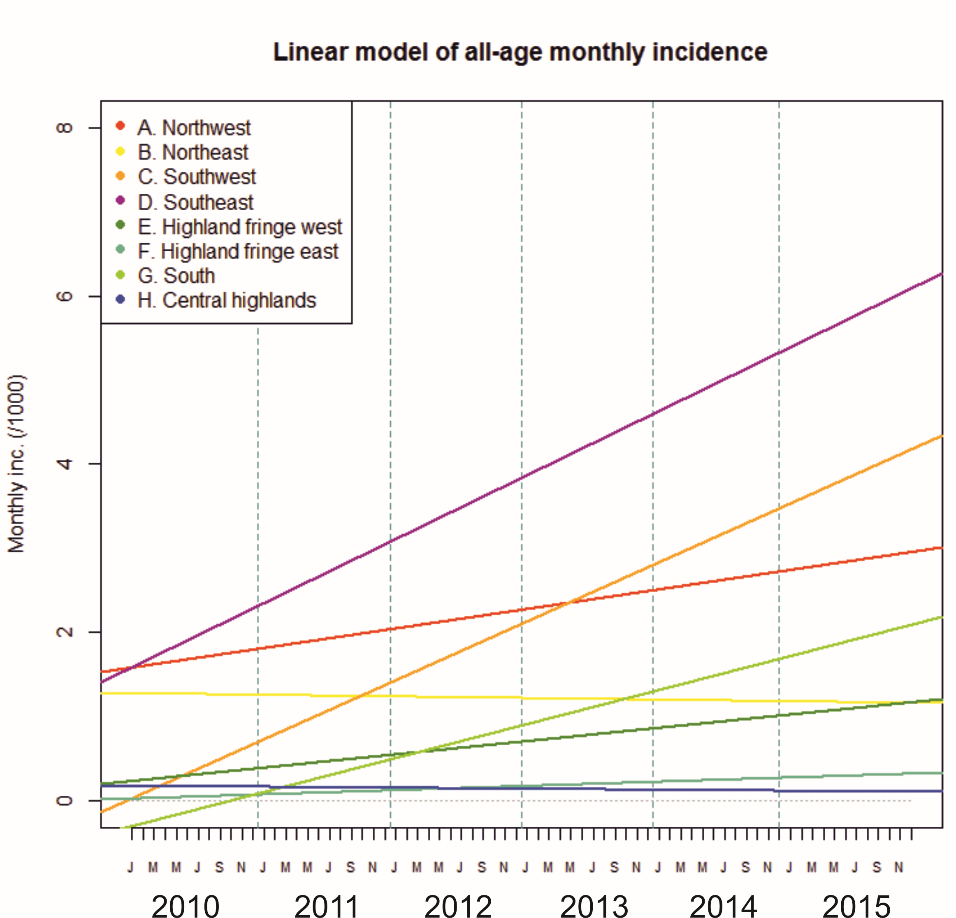


**Figure S2.4.B. Summary plot of the linear models from each ecozone**. Summary statistics are presented in Table S2.1.

| **Ecozone** | **Effect size (β) of change in monthly all-age incidence**  **(2010-2015)** | **P-value** |
| --- | --- | --- |
| 1. Northwest | 0.019468 | 0.00265 |
| 1. Northeast | -0.001552 | 0.635 |
| 1. Southwest | 0.058440 | 5.11e-09 |
| 1. Southeast | 0.06356 | 9.74e-05 |
| 1. Highland fringe west | 0.01314 | 0.0016 |
| 1. Highland fringe east | 0.0041178 | 1.29e-05 |
| 1. South | 0.033561 | 8.48e-07 |
| 1. Central Highlands | -0.0009594 | 0.0712 |

**Table S2.1. Summary statistics of the linear models of each ecozone’s time series of case incidence (2010-2015).** Significant P-values are highlighted in red.

1. **Reported contribution of confirmed malaria cases to overall health facility consultations.**

These plots summarise the proportion of health facility consultations resulting in an RDT+ diagnosis, indicating the relative burden of malaria on the health system over time.

The monthly reports submitted by each health facility to their respective health districts include both the total number of consultations that took place and the numbers of RDT+ cases diagnosed. The proportion of consultations attributable to malaria each month provides insight into the trends of malaria burden, independent of the potential influence of under-reporting.


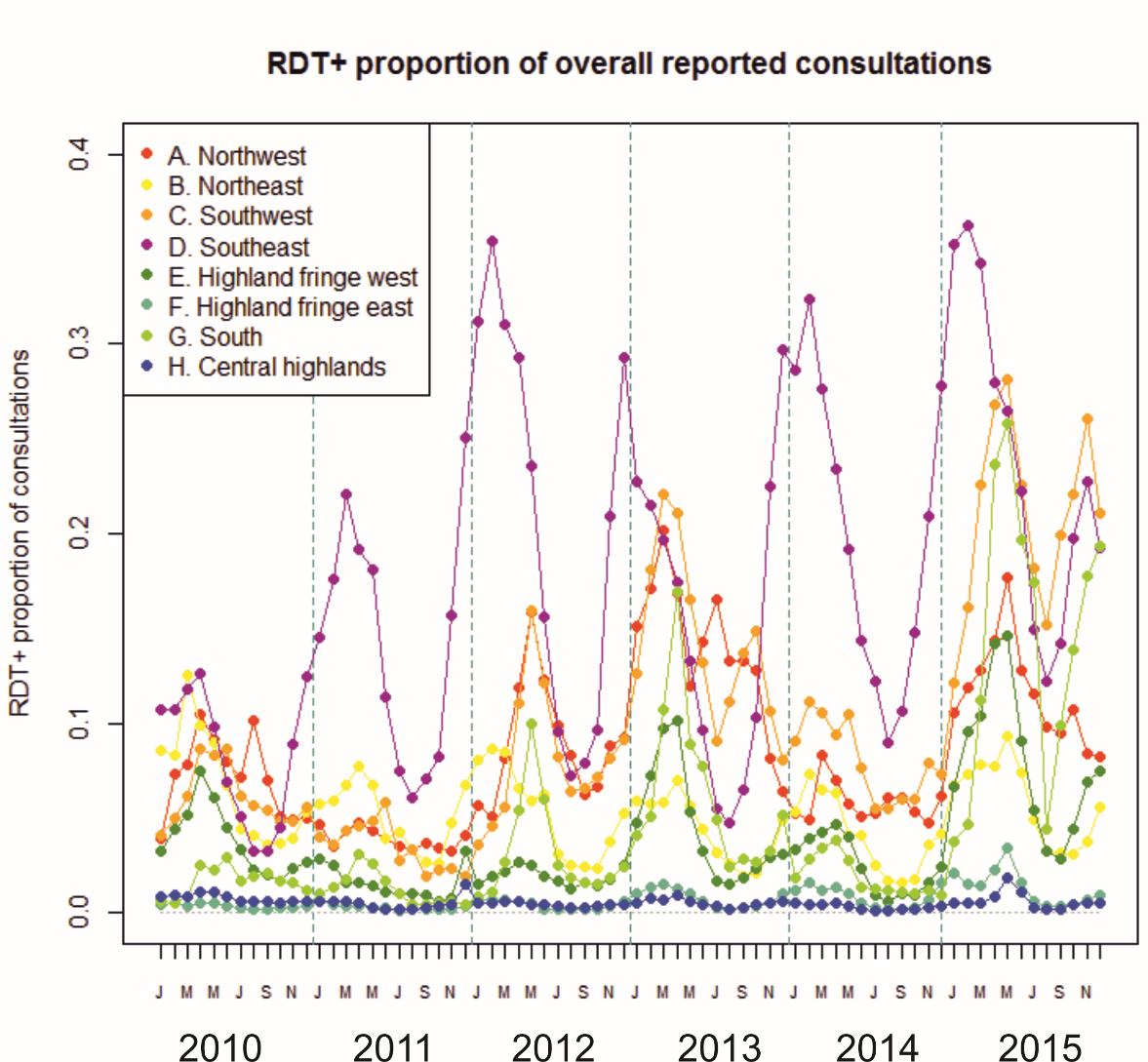


**Figure S2.5. Proportion of all consultations reported monthly from each ecozone resulting in an RDT+ diagnosis.** All ecozone data are plotted together to allow relative comparisons between areas.


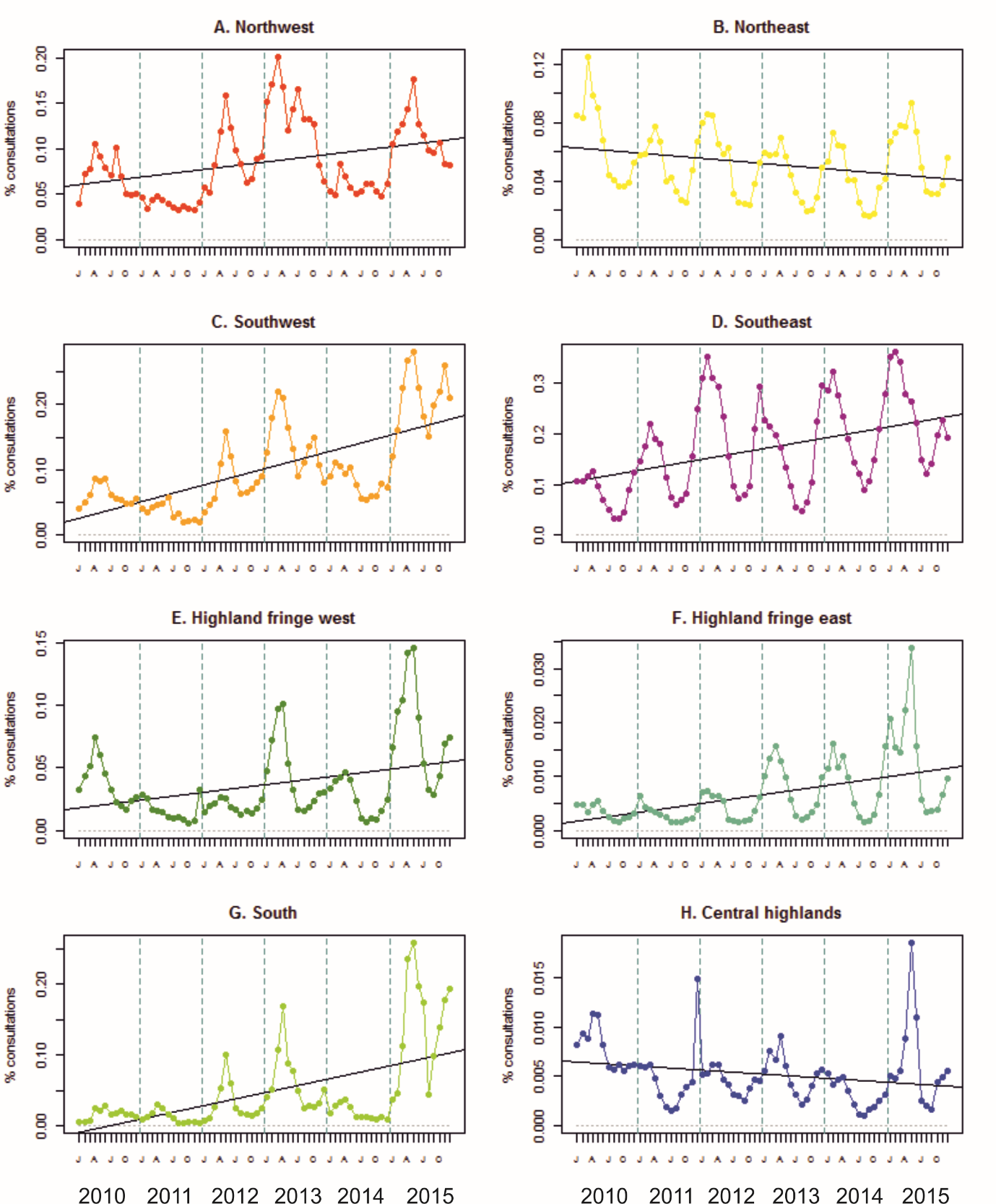


**Figure S2.6. Linear models of the time series of the proportion of RDT+ consultations (2010-2015).** The linear regression distils the intra-annual monthly fluctuations to reveal overall trends over time. Linear model significance is shown in Table S2.2.


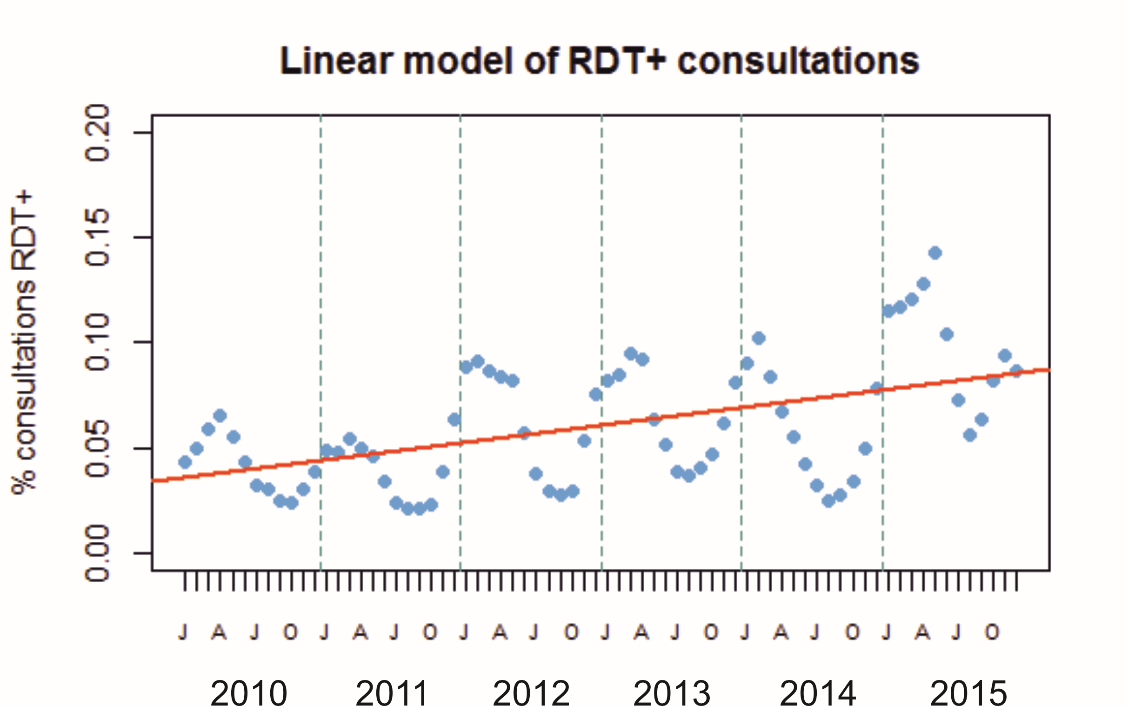


**Figure S2.7. National-level linear regression model of the proportion of health facility consultations that were RDT+ over the 2010-2015 time series**. Model parameters are listed in Table S2.2.

| **Ecozone** | **Effect size (β) of monthly change in proportion of RDT+ consultations (2010-2015)** | **P-value** |
| --- | --- | --- |
| Madagascar (all ecozones) | 0.069% | 7.32e-06 |
| 1. Northwest | 0.068% | 0.00321 |
| 1. Northeast | -0.031% | 0.0178 |
| 1. Southwest | 0.212% | 1.18e-10 |
| 1. Southeast | 0.181% | 0.000264 |
| 1. Highland fringe west | 0.052% | 0.00212 |
| 1. Highland fringe east | 0.014% | 2.13e-05 |
| 1. South | 0.156% | 3.39e-07 |
| 1. Central Highlands | -0.0003% | 0.0518 |

**Table S2.2. Summary statistics of the linear models of each ecozone’s time series of the proportion of health-centre consultations that were RDT+ during the 2010-2015 period.** Significant P-values (p<0.05) are highlighted in red.

These results indicate a significant increase in the contribution of malaria to overall health facility consultations. The model estimate of 0.069% monthly increases nationally equates to an overall 4.14% increase over the 2010-2015 reporting period, corresponding to a more than doubling estimated by the fitted linear model across the time series (from 3.6% model estimate at time 0 to 8.5% at month 72). The results in Table S2.2 indicate that all ecozones have reported a significant increase in the proportion of consultations attributed to a malaria diagnosis, except the Northeast where there has been a decrease over the six year reporting period. The increase was greatest in the Southwest ecozone, where there was a 0.2% increase each month in the contribution of malaria to overall contributions. This corresponds to a 2.5% annual increase, or a 15.3% increase across the 2010-2015 time period. Reports from the Central Highlands indicate a decrease in malaria burden to health facilities, but this is not statistically significant.

1. **RDT positivity rate**

The RDT positivity rate is an indicator of the contribution of malaria to overall numbers of fevers. This metric is less sensitive to reporting rates, diagnostic kit availability, and treatment-seeking behaviour as it refers only to the proportion of tests conducted which yielded a positive result [[1](#_ENREF_1)]. The metric allows comparisons to be made between different ecozones independent of the potential confounders that the other metrics discussed so far are vulnerable to (A. Raw reported numbers of RDT+ cases; B. Incidence of infection; C. Proportion of RDT+ consultations).


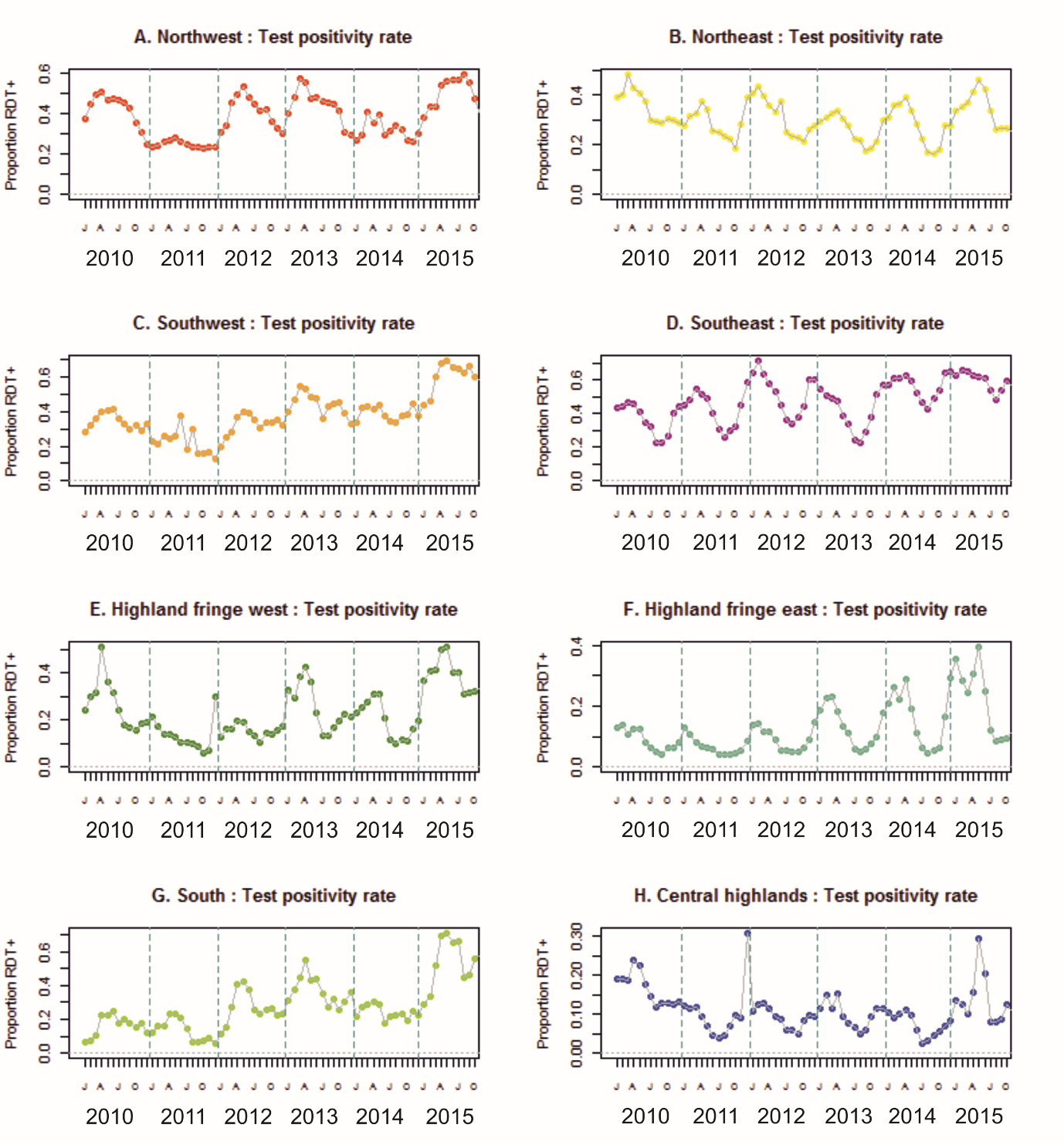


**Figure S2.8. Test positivity rates by ecozone (2010-2015).** This metric is calculated as the proportion of all rapid diagnostic tests for malaria that have a positive result.


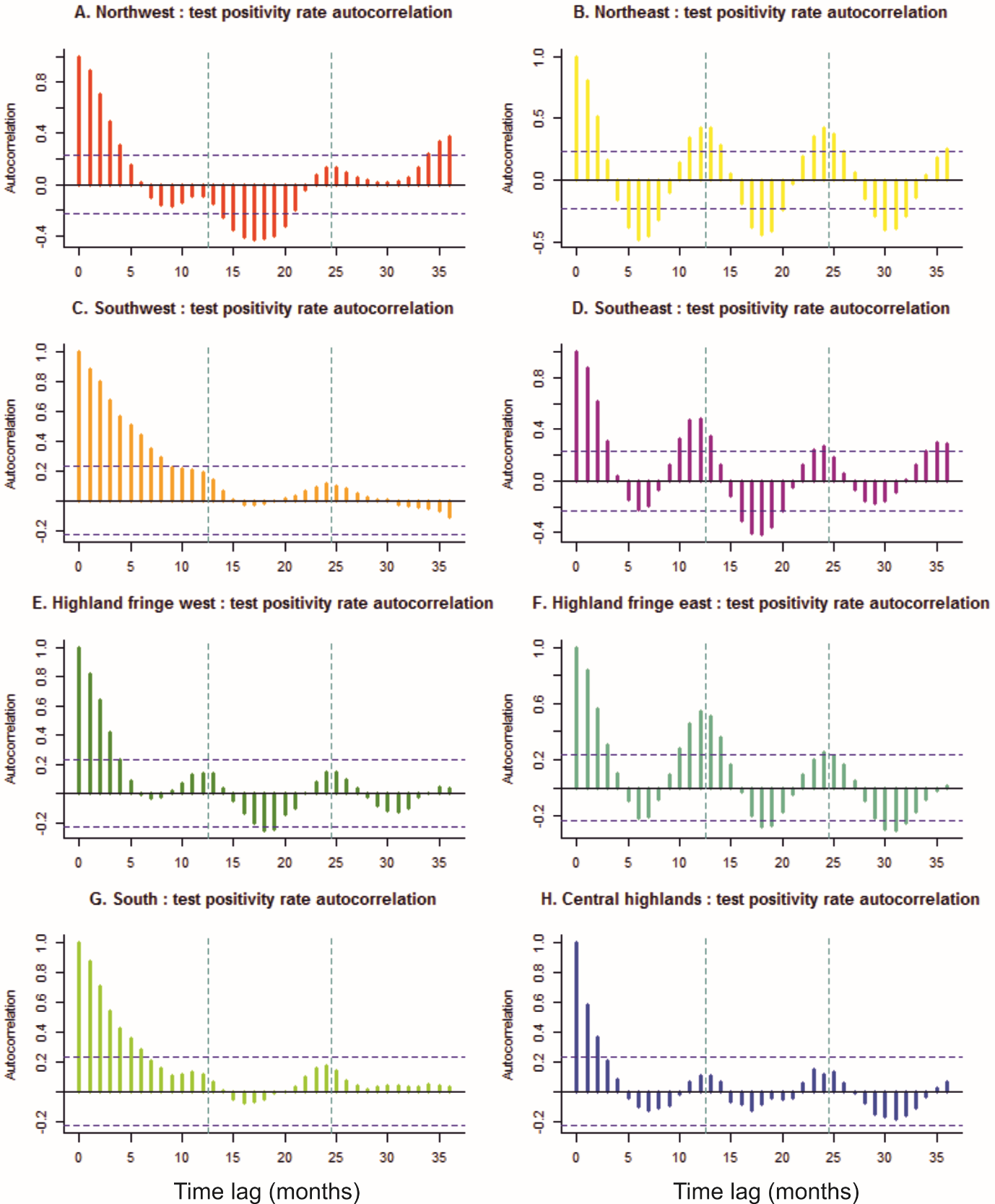


**Figure S2.9. Autocorrelation in the time series of test positivity rates across the 2010-2015 time period**.

The diagnostic positivity rates reveal similar autocorrelation to the correlations across the case incidence time series (Figure S2.2). Correlations with annual periodicity (lag time of 12 months) are apparent from the eastern ecozones (B. Northeast; D. Southeast; F. Highland fringe east).

**
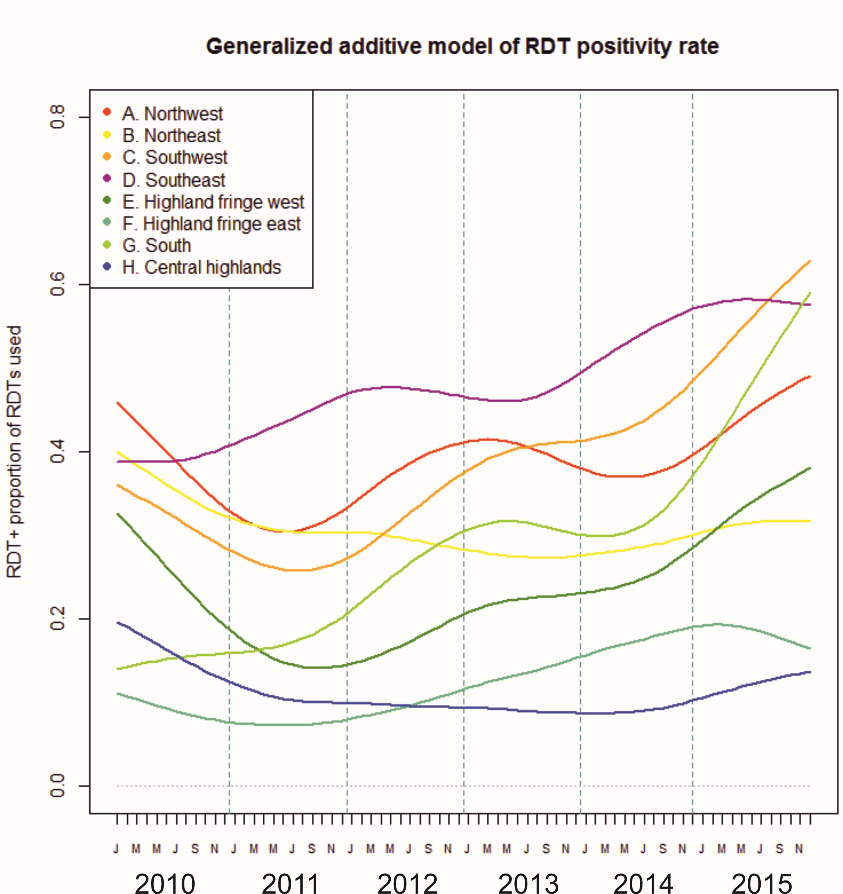
**

**Figure S2.10 Generalized additive models of the change in RDT positivity rate over time by ecozone**. The model was set to use a smoothing effect of 4 months. A smoothing value of 4 was set. This smoothing model facilitates comparisons of temporal trends between ecozones


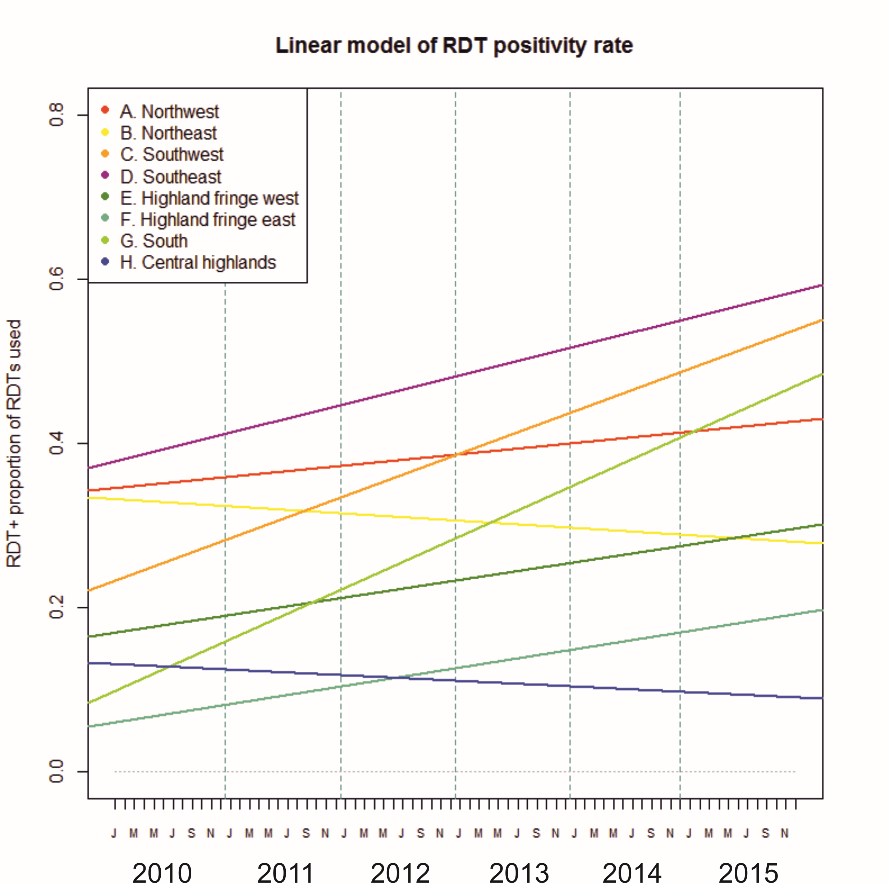


**Figure S2.11A. Linear regression models of the change in RDT positivity rate over time by ecozone.** Model terms are shown in Table S2.3.

**
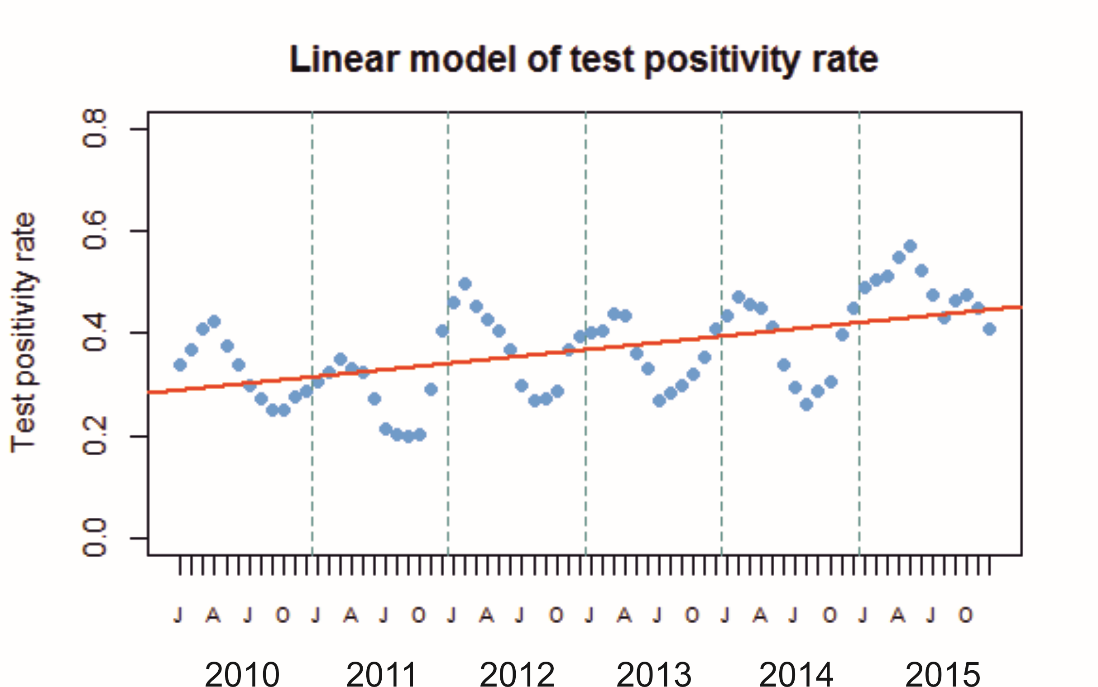
**

**Figure S2.11B. Linear regression model of the time series of national-level test positivity rate.** Model summary statistics are given in Table S2.3.

| **Ecozone** | **Effect size (β) of monthly change in RDT positivity rate**  **(2010-2015)** | **P-value** |
| --- | --- | --- |
| Madagascar (all ecozones) | 0.224% | 2.14e-06 |
| 1. Northwest | 0.116% | 0.0517 |
| 1. Northeast | -0.075% | 0.0756 |
| 1. Southwest | 0.430% | 1.99e-11 |
| 1. Southeast | 0.291% | 1.38e-05 |
| 1. Highland fringe west | 0.179% | 0.00449 |
| 1. Highland fringe east | 0.185% | 3.41e-05 |
| 1. South | 0.522% | 2.13e-11 |
| 1. Central highlands | -0.058% | 0.0605 |

**Table S2.3. Summary statistics of the linear models of temporal trends in diagnostic test positivity rates (2010-2015).** Significant P-values (p<0.05) are highlighted in red. The effect size of the change (β) in RDT positivity rate is shown by month. The sharpest rate of increase is therefore in the South ecozone. Two ecozones (B. Northeast and H. Central highlands) report a negative trend, though this is not statistically significant.


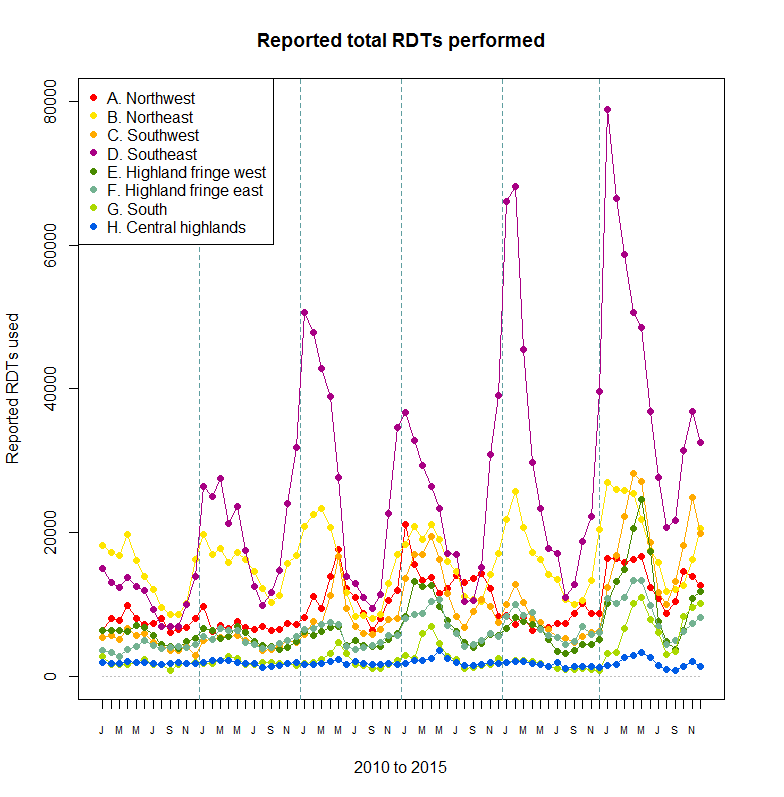


**Figure S2.12 Barplot of total RDTs performed.** The data plotted here shows the monthly trends across ecozones in the total numbers of RDTs performed. Trends in reported RDT use are a function of numbers of fevers presenting at clinics and of RDT availability.

**Reference**

1. WHO: **Disease Surveillance for Malaria Control: an Operational Manual.** Geneva, Switzerland; 2012.
